# Preclinical efficacy of a cell division protein candidate gonococcal vaccine identified by artificial intelligence

**DOI:** 10.1128/mbio.02500-23

**Published:** 2023-10-31

**Authors:** Sunita Gulati, Andreas Holm Mattsson, Sophie Schussek, Bo Zheng, Rosane B. DeOliveira, Jutamas Shaughnessy, Lisa A. Lewis, Peter A. Rice, Pär Comstedt, Sanjay Ram

**Affiliations:** 1 Department of Medicine, Division of Infectious Diseases and Immunology, University of Massachusetts Medical School, Worcester, Massachusetts, USA; 2 EVAXION Biotech, Hørsholm, Denmark; Albert Einstein College of Medicine, Bronx, New York, USA

**Keywords:** *Neisseria gonorrhoeae*, vaccine, complement, artificial intelligence

## Abstract

A safe and effective vaccine is urgently needed to combat the global threat of multidrug-resistant (MDR) *Neisseria gonorrhoeae*. We screened 26 gonococcal proteins discovered by an artificial intelligence-driven platform called Efficacy Discriminative Educated Network (EDEN) trained to identify novel, protective vaccine antigens against pathogenic bacteria for efficacy in the mouse vaginal colonization model of gonorrhea. Combinations of two to three antigens adjuvanted with GLA-SE (induces T_H_1 responses) yielded 11 groups that were used to vaccinate mice. An inverse correlation was noted between the complement-dependent bactericidal activity of antisera from each of the 11 groups and the burden of gonococcal colonization. The combination of NGO1549 (FtsN; cell divisome protein) and NGO0265 (predicted cell division protein) most substantially reduced the burden of colonization by MDR strain WHO X. The EDEN prediction score for each group of antigens correlated positively with reductions in overall bacterial burden, providing evidence for its predictive potential. FtsN and NGO0265 administered either individually, in combination, or as a chimeric protein significantly attenuated gonococcal vaginal colonization by all three test strains. IgG in antisera from mice immunized with the chimeric NGO0265-FtsN protein supported the complement-dependent killing of all 50 (100%) gonococcal isolates tested. The efficacy of the chimeric NGO0265-FtsN vaccine required the membrane attack complex (C5b-9) of complement, evidenced by loss of efficacy in complement *C9^−/−^* mice. In conclusion, a chimeric molecule comprising NGO0265 and FtsN adjuvanted with GLA-SE elicits IgG with broad anti-gonococcal bactericidal activity, attenuates gonococcal colonization in a complement-dependent manner, and represents a promising gonococcal vaccine candidate.

**IMPORTANCE:**

Vaccines to curb the global spread of multidrug-resistant gonorrhea are urgently needed. Here, 26 vaccine candidates identified by an artificial intelligence-driven platform (Efficacy Discriminative Educated Network[EDEN]) were screened for efficacy in the mouse vaginal colonization model. Complement-dependent bactericidal activity of antisera and the EDEN protective scores both correlated positively with the reduction in overall bacterial colonization burden. NGO1549 (FtsN) and NGO0265, both involved in cell division, displayed the best activity and were selected for further development. Both antigens, when fused to create a chimeric protein, elicited bactericidal antibodies against a wide array of gonococcal isolates and significantly attenuated the duration and burden of gonococcal colonization of mouse vaginas. Protection was abrogated in mice that lacked complement C9, the last step in the formation of the membrane attack complex pore, suggesting complement-dependent bactericidal activity as a mechanistic correlate of protection of the vaccine. FtsN and NGO0265 represent promising vaccine candidates against gonorrhea.

## INTRODUCTION

*Neisseria gonorrhoeae*, the causative agent of the sexually transmitted infection gonorrhea, has developed resistance to almost every antibiotic in clinical use (1, 2). Ceftriaxone is now the only recommended first-line treatment for gonorrhea (3). Previously, azithromycin together with ceftriaxone was recommended for the treatment of gonorrhea to delay the development of resistance and to also treat chlamydia co-infection. However, rates of resistance of *N. gonorrhoeae* to azithromycin exceed 5% and it is, therefore, no longer recommended for treating gonorrhea (3). Ceftriaxone resistance has also been reported from various parts of the world. In addition, the number of cases of gonorrhea continues to climb—in 2021, a total of 710,151 cases of gonorrhea were reported to the CDC, which represent a 118% increase since the historic low in 2009 (4). Consequently, there is an urgent need to develop safe and effective vaccines against gonorrhea.

Clinical trials carried out prior to 1990 employed killed whole cells, pili, or outer membrane preparations enriched in porin (PorB) but were unsuccessful and slowed further gonococcal vaccine development efforts (2). However, retrospective epidemiologic studies in recipients of detergent-extracted group B meningococcal outer membrane vesicle vaccines (OMVs) have shown a 31%–46% reduction in gonococcal infection; protection was greatly reduced in patients co-infected with chlamydia (5–8). These observations have provided a reason for optimism for gonococcal vaccine development. The past decade has seen several gonococcal vaccine candidates at various stages of preclinical development including transferrin binding protein B (TbpB), Neisserial Heparin Binding Antigen (NHBA), D-methionine binding protein (MetQ), meningococcal OMVs, intranasally or intravaginally gonococcal outer membrane vesicles in combination with IL-12 slow-releasing microspheres and a peptide mimic of a lipooligosaccharide (LOS) glycan epitope recognized by monoclonal antibody 2C7 (2, 9–17).

An artificial intelligence (AI) platform, Efficacy Discriminative Educated Network (EDEN; EVAXION Biotech), was developed to identify potential vaccine candidates *in silico*. Here, we evaluated 26 antigens identified by EDEN for their efficacy against *N. gonorrhoeae* and have identified two novel antigens with promising efficacy in pre-clinical studies.

## RESULTS

### Derivation of gonococcal vaccine candidate antigens using the EDEN bacterial antigen discovery platform

The genome sequences of 10 strains of *N. gonorrhoeae*, FA1090, FDAARGOS_207, NCCP11945, FDAARGOS_205, 34530, 32867, 34769, FDAARGOS_204, MS11, and H041 (WHO X), were used to derive proteomes that were processed through EDEN. The Cross Protection Score (CPS) for each protein was calculated based on strain-specific EDEN prediction score and homology conservation across the first nine complete genome-sequenced strain proteomes, using FA1090 as the primary strain. A total of 26 proteins out of 1,883 processed proteins comprised in the primary FA1090 proteome with the highest CPS were selected as top rank EDEN-predicted vaccine antigens.

### Efficacy of recombinant gonococcal antigens adjuvanted with GLA-SE

**Fig 1:**
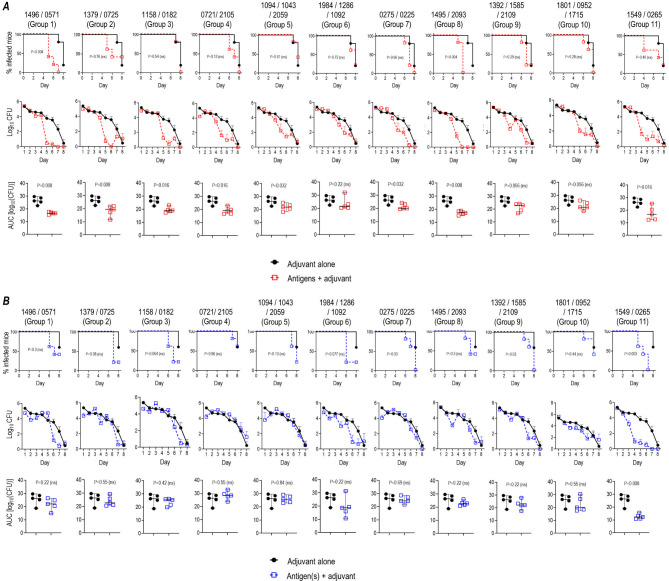
Efficacy of 26 gonococcal vaccine antigens against *N. gonorrhoeae* strains MS11 (**A**) and H041 (WHO X) (**B**) in the mouse vaginal colonization model. Mice were immunized with combinations of individual antigens (15 µg of each antigen) indicated at the top of each column in the graphs (corresponding NGO numbers, indicated above each column); vaccines were adjuvanted with either 5 µg GLA-SE (groups 1–6 and group 11) or 5 µg GLA-SE plus Al(OH)_3_ (groups 7–10). Mice were immunized intramuscularly at weeks 0, 3, and 6. Adjuvant control mice received GLA-SE alone. Two weeks post-dose 3, mice in the diestrus phase of the estrous cycle (*n* = 5/group) were challenged intravaginally with *N. gonorrhoeae* MS11 (2.6 × 10^7^ CFU) (**A**) or H041 (WHO X) (3.8 × 10^7^ CFU) (**B**). Vaginas were swabbed daily to enumerate gonococcal CFUs. In panels **A** and **B**, the top rows show times to clearance (Kaplan–Meier curves; groups compared by Mantel-Cox analysis), the middle rows show log_10_ CFU versus time, and the bottom rows show analysis of AUC (means, ±95% confidence intervals were compared across groups by Mann Whitney’s non-parametric test).

Previous studies have suggested that Th1-biased responses are important for protecting vaccinated mice against gonorrhea (18, 19). Therefore, we used GLA-SE, a TLR4 agonist that induces Th1-skewed responses (20, 21). We tested the efficacy of 26 gonococcal vaccine candidate antigens (described in Table S1) adjuvanted with GLA-SE. The putative location of these proteins in the cell was determined by PSORTb (Table S2). The antigens were tested in groups of two to three antigens each to accommodate all antigens in a single protection experiment. Proteins with similar CPS ranks were grouped together with the assumption that groups with higher average CPS scores would show greater efficacy than groups with lower average CPS scores. The antigens in groups 7, 8, 9, and 10 were in urea and, therefore, washed in phosphate-buffered saline (PBS) in the presence of alum to remove urea and facilitate refolding of the protein on alum, followed by the addition of GLA-SE. Protective efficacy in mice vaccinated with each of the 11 groups of antigens was tested against challenge with strains MS11 (Fig. 1A) and H041 (WHO X) (Fig. 1B). Except for two groups, 6 and 9 (NGO1984/NGO1286/NGO1092 and NGO1392/NGO1585/NGO2109, respectively), which did not show efficacy in the Kaplan–Meier time to clearance analysis (Fig. 1A, top row) or area under the curve (AUC) analysis (Fig. 1A, bottom row), all other groups showed activity against MS11 challenge by at least one of these two parameters. In animals challenged with H041, efficacy was noted only in the group immunized with the combination of NGO1549 (FtsN; a component of the gonococcal divisome) and NGO0265 (predicted to be involved in cell division).

**Fig 2:**
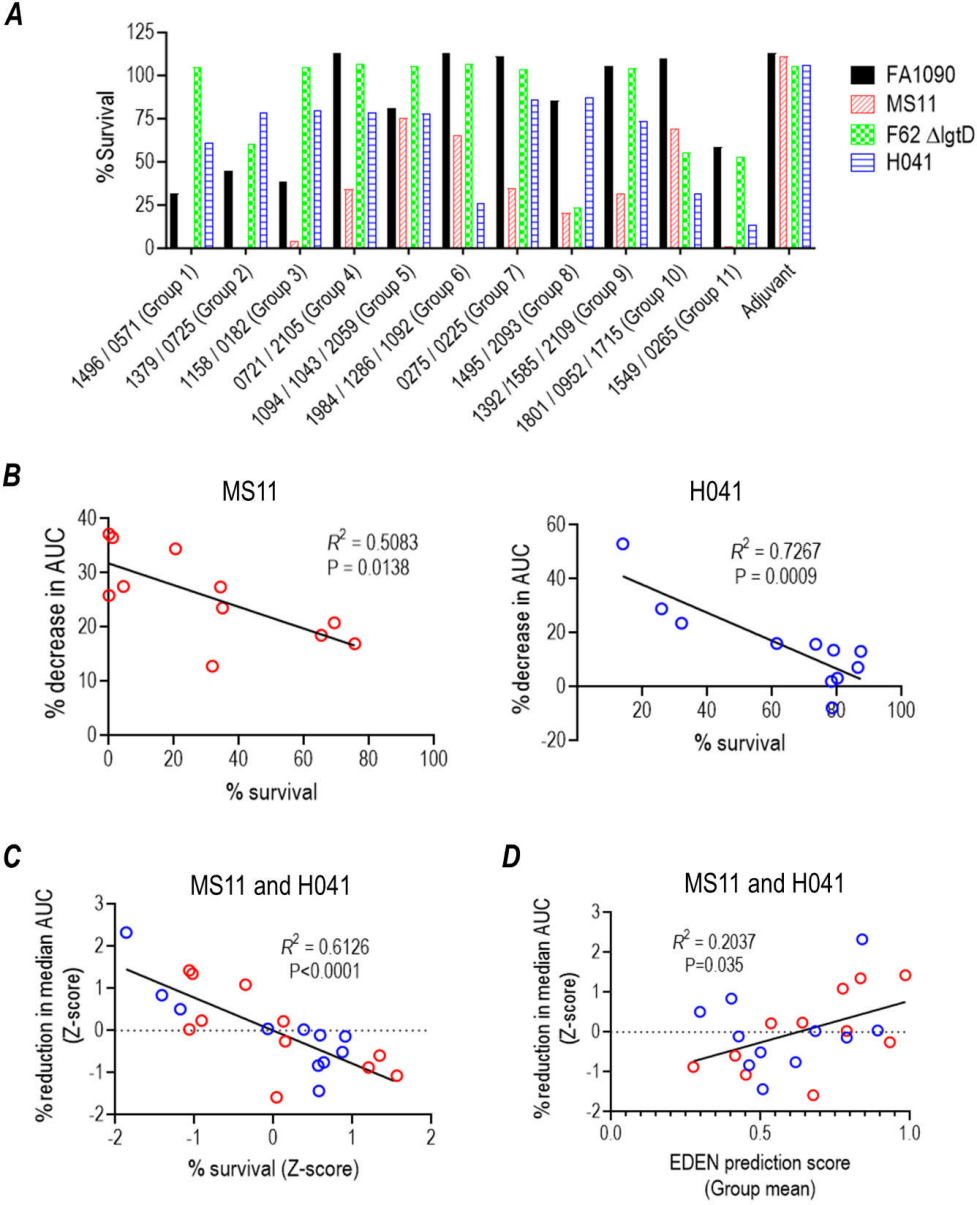
Bactericidal activity of IgG in antisera from the 11 groups of mice immunized with 26 gonococcal antigens and correlation with efficacy in mice. (**A)** Complement-dependent bactericidal activity of IgG in sera from 11 groups of mice immunized with 26 gonococcal antigens against four strains of *N. gonorrhoeae*. Sera from 10 mice, immunized but not challenged with *N. gonorrhoeae*, were pooled, depleted of mouse IgM, and incubated with strains FA1090, MS11, F62 ΔlgtD, and H041. IgG- and IgM-depleted normal human serum at a final concentration of 28% (or 11% for MS11) was used as a source of complement. CFUs at 30 min relative to 0 min are expressed as percentage as shown on the *Y*-axis. (**B)** Survival of MS11 and H041 in serum bactericidal assays correlates inversely with the percent reduction in AUC in the mouse vaginal colonization model. The percent reduction in median AUCs of immunized mice versus adjuvant control mice was plotted against percent survival in serum bactericidal assays. Data were analyzed using linear regression (note that one datum point in the H041 graph lies beneath the *X*-axis and is superimposed on the “90” *X*-axis label). (**C)** Correlation between the survival of MS11 (red circles) and H041 (blue circles) in serum bactericidal assays and percent reduction in median AUCs using *Z*-score values for both parameters. *Z*-scores for each data point were calculated using the formula *Z* = (*X* − µ)/σ, where *X* is the raw (non-normalized) value, and µ and σ are the mean and standard deviations, respectively of each of the four sets of data (survival in bactericidal assays and percent reduction in median AUC for each strain). (**D)** Correlation between strain-specific EDEN prediction scores (group mean) and relative reduction in median AUCs (*Z*-scores) for MS11 (red circles) and H041 (blue circles).

Immune sera from vaccinated mice not challenged with *N. gonorrhoeae* were pooled, depleted of IgM to eliminate killing by naturally occurring bactericidal IgM against gonococci as reported previously (16), and tested for bactericidal activity against four gonococcal strains (FA1090, MS11, F62 ΔlgtD, and H041) (Fig. 2A). The ΔlgtD mutant of F62 lacks the terminal GalNAc beyond HepI lacto-*N*-neotetraose, a target for bactericidal IgM in human serum (22, 23); the use of F62 ΔlgtD eliminated reduced survival of wild-type (WT) F62 caused by residual IgM in some batches of human complement. A higher concentration of immune serum (56%) was used to enhance the sensitivity of the assay. Killing varied across groups of sera and strains; MS11 was the most sensitive, and with the exception of three pools of serum (groups 5, 6, and 10), it was killed >50% (<50% bacterial survival) by all sera. Only groups 6, 10, and 11 antisera killed H041 (WHO X) >50% (i.e., <50% survival). FA1090 was killed >50% by antisera from groups 1, 2, and 3, while F62 ΔlgtD was killed >50% only by group 8 antiserum. The immunogenicity of each immune serum pool against the immunizing antigen and/or lysates from four strains (FA1090, MS11, F62 ΔlgtD, and H041) is shown in Fig. S1.

An inverse correlation between bacterial survival in bactericidal assays and the percent reduction in median AUC compared to the adjuvant control group was observed for both MS11 and H041 (linear regression; Fig. 2B). When data from each of the experiments were normalized using *Z*-scores (Table S3A and B list the EDEN prediction scores, AUCs, and bactericidal data, and the derived *Z*-scores for strains MS11 and H041 [WHO X], respectively), a strong correlation was observed between survival in bactericidal assays *in vitro* and *in vivo* protection measured as reductions in AUC (Fig. 2C), suggesting complement-dependent killing as a probable mechanistic correlate of protection in the mouse vaginal colonization model. Furthermore, the EDEN predictive score correlated directly with the AUC *Z*-scores for MS11 and H041 (Fig. 2D), providing evidence of its predictive potential.

**Fig 3:**
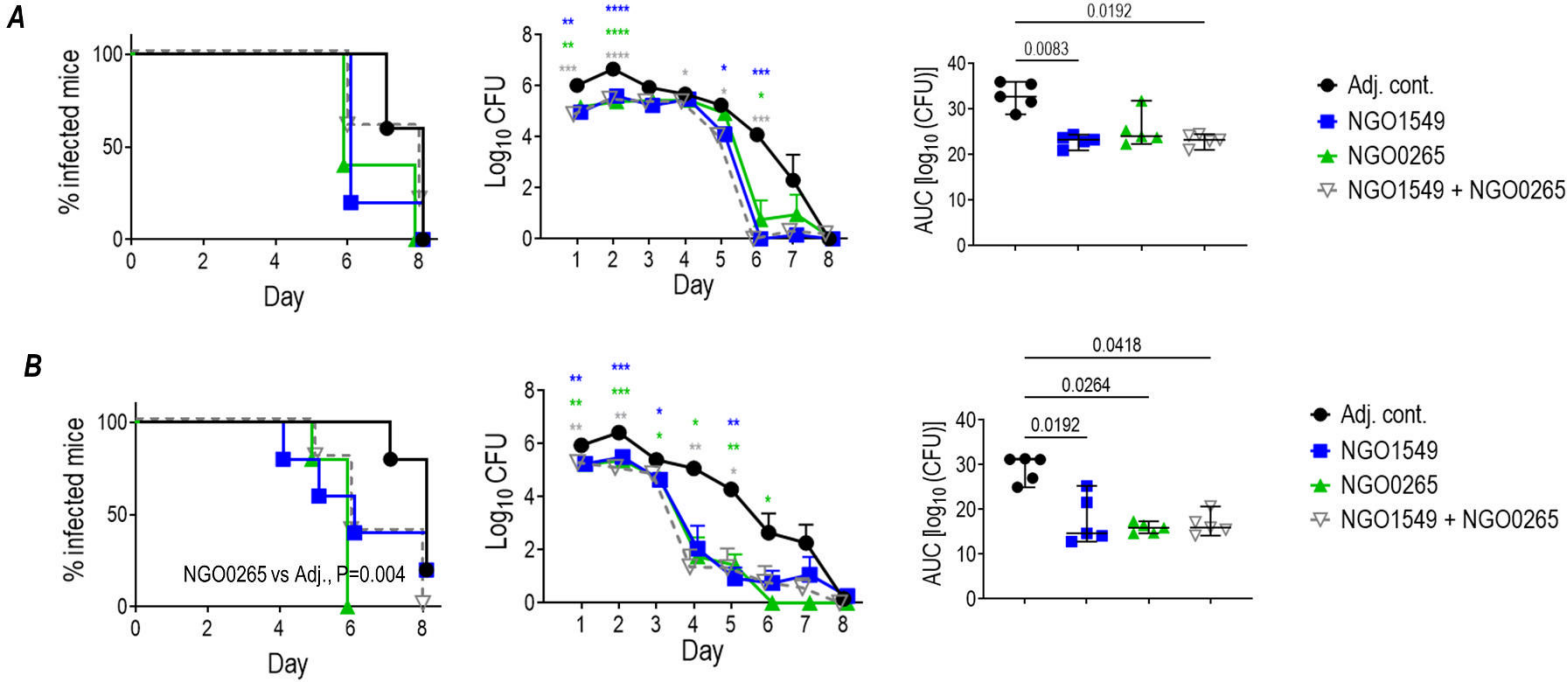
Efficacy of NGO0265 and NGO1549 either singly or in combination against *N. gonorrhoeae* strains FA1090 and MS11. Female BALB/c mice were immunized with NGO1549 or NGO0265 (15 µg of each antigen, either alone or in combination) adjuvanted with GLA-SE (5 µg), intramuscularly at 0, 2, and 4 weeks. Adjuvant controls (Adj. cont.) received GLA-SE alone. Two weeks post-dose 3, mice were challenged with either strain FA1090 (3.6 × 10^7^ CFU/mouse; *n* = 5/group) (**A**) or MS11 (2.8 × 10^7^ CFU/mouse; *n* = 5/group) (**B**). Vaginas were swabbed daily to enumerate gonococcal CFUs. Kaplan–Meier curves showing time to clearance (graphs on left). Graphs in the middle column show log_10_ CFU versus time. Comparisons were made by two-way ANOVA and comparisons between each vaccine group with the control group on each day by Dunnett’s multiple comparisons test. *P < 0.05; **P < 0.01; ***P < 0.001; and ****P < 0.0001. The colors of the asterisks correspond to the color of the line of the vaccine group. Graphs on the right show AUC (mean, ±95% confidence intervals). Comparisons across groups were made by one-way ANOVA using the non-parametric Kruskal-Wallis equality of populations rank test. The Kruskal-Wallis statistics were 10.98 (*P* = 0.0119) and 10.22 (*P* = 0.0168) for the graphs in panels **A** and **B**, respectively. AUC comparisons between each group and the adjuvant control group were made using Dunn’s multiple comparisons test.

The efficacy of NGO0265 and NGO1549, either alone or in combination, was tested against strains MS11 and FA1090 (Fig. 3). In this experiment, the combination of both proteins was not more effective than either protein used alone. Pooled serum from each of the groups again showed similar killing against the panel of four strains: strains MS11 and H041 were susceptible (>50% killing), while FA1090 and F62 ΔlgtD showed intermediate susceptibility (50%–75% survival) (Fig. S2). The expression levels of both proteins were similar across the four strains by western blotting (Fig. S3A), suggesting that differences in the amounts of NGO0265 and NGO1549 expressed likely did not account for differences in bactericidal activity across the four isolates. We noted the binding of antisera against NGO1549 (FtsN) and NGO0265 to strain H041 (WHO X) by flow cytometry (Fig. S3B), suggesting that immune antibodies could bind to both proteins on intact bacteria.

### Efficacy of a chimeric antigen comprising NGO0265 and NGO1549

**Fig 4:**
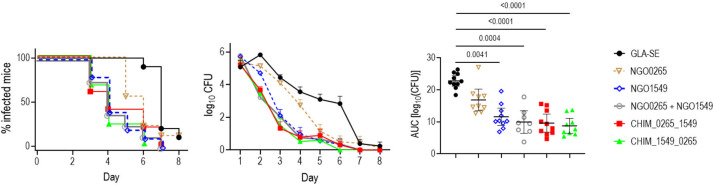
Efficacy of NGO1549 and NGO0265 chimeric proteins adjuvanted with GLA-SE against *N. gonorrhoeae* in mice. Six-week-old female BALB/c mice were immunized with NGO0265, NGO1549 (FtsN), a combination (mixture) of NGO0265 and NGO1549, a chimera of NGO0265 (N-terminal) and NGO1549 (C-terminal) (CHIM_0265_1549), or a chimera with NGO1549 (N-terminal) and NGO0265 (C-terminal) (CHIM_1549_0265). All formulations were adjuvanted with GLA-SE (5 µg/dose). NGO0265 and NGO1549 (FtsN) were each used at 15 µg/dose when given individually or in combination; chimeric proteins were administered at 25 µg/dose. Adjuvant control mice received GLA-SE alone. Mice were immunized intramuscularly at 0, 3, and 6 weeks and challenged intravaginally with *N. gonorrhoeae* H041 (WHO X) (6 × 10^7^ CFU) 2 weeks post-dose 3 (*n* = 10 mice per group). Vaginas were swabbed daily to enumerate CFUs. Left graph: time to clearance (Kaplan–Meier curves, analyzed by Mantel-Cox log-rank test). *P* values for the NGO0265, NGO1549, NGO0265 plus NGO1549, CHIM_0265_1549, and CHIM_1549_0265 versus the adjuvant control group were 0.04, 0.0001, 0.0003, 0.0009, and <0.0001, respectively. Middle graph: log_10_ CFU versus time. Right graph: AUC (mean, ±95% confidence intervals). Comparisons across groups were made by one-way ANOVA using the non-parametric Kruskal-Wallis equality of populations rank test. The Kruskal-Wallis statistic was 33.8 (*P* = 0.0001). AUC comparisons between each group and the adjuvant control group were made using Dunn’s multiple comparisons test.

Given the efficacy of NGO0265 and NGO1549 when used singly, we evaluated chimeric antigens comprising both proteins. Targeting both proteins could increase the breadth of protection, increase the barrier for the development of resistance, and/or provide greater bactericidal activity by increasing antibody density on the bacterial surface. Two chimeric proteins, CHIM_0265_1549 (NGO0265 located N-terminal) and CHIM_1549_0265 (NGO1549 located N-terminal), where the protein subunits were separated by a GSGGG linker, were constructed. NGO1549 and NGO0265 alone, or in combination (as a mixture) were used as comparators. GLA-SE was used as an adjuvant. Two weeks post-dose 3, mice were challenged with strain H041 (WHO X). Both chimeric molecules, NGO1549 and the combination of NGO1549 and NGO0265, showed similar levels of efficacy (Fig. 4). Although protection induced with NGO0265 alone in this experiment was not statistically significant, a 33% reduction in median AUC compared to the adjuvant control group was noted.

**Fig 5:**
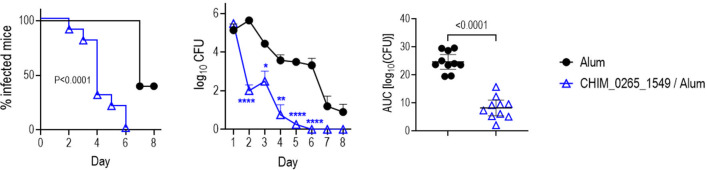
Chimeric protein CHIM_0265_1549 adjuvanted with aluminum hydroxide (Alhydrogel) effectively clears gonococcal vaginal colonization in mice. Six-week-old female BALB/c mice were immunized with CHIM_0265_1549 (25 µg) adjuvanted with Al(OH)_3_ (Alhydrogel) intramuscularly at weeks 0, 3, and 6. Two weeks post-dose 3, mice were challenged intravaginally with *N. gonorrhoeae* H041 (WHO X). Vaginas were swabbed daily to enumerate CFUs. Left graph: Kaplan–Meier curves showing time to clearance, analyzed by Mantel-Cox log-rank test. Middle graph: log_10_ CFU versus time, analyzed by two-way ANOVA (with Šídák’s multiple comparisons test). *P < 0.05; **P < 0.01; and ****P < 0.0001. Right graph: AUC analysis (mean, ±95% confidence intervals). Comparisons were made with Mann-Whitney’s test.

**Fig 6:**
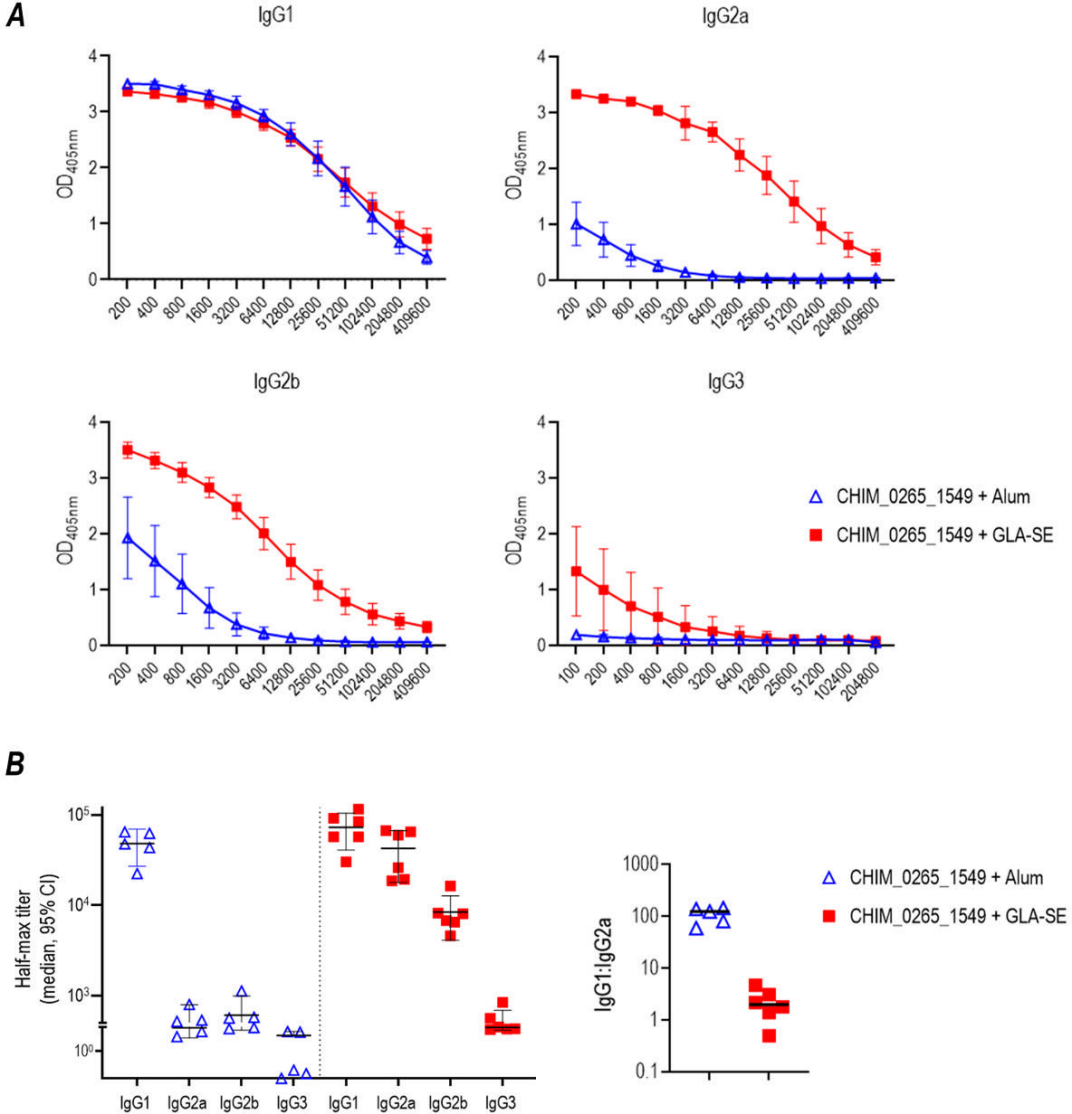
GLA-SE and alum elicit T_H_1 and T_H_2-biased responses, respectively. (**A)** IgG subclasses elicited by CHIM_0265_1549 adjuvanted with either alum or GLA-SE. IgG subclasses in sera from mice immunized with CHIM_0265_1549/GLA SE (red line/solid red squares; *n* = 6) or CHIM_0265_1549/alum (blue line/blue triangles; *n* = 5) were measured by ELISA. *X*-axis, reciprocal serum dilution; *Y*-axis, absorbance (OD_405nm_). (**B)** The left graph shows half-maximum (Half-max) titers (calculated dilution that yields half-maximal OD_405nm_; median [95% CI]). The graph on the right shows IgG1:IgG2a ratios for each mouse, calculated using Half-max titers (median).

The experiments thus far all used GLA-SE, a T_H_1-biased adjuvant. Therefore, we next tested the efficacy of CHIM_0265_1549 adjuvanted with Alhydrogel [Al(OH)_3_], which usually elicits a T_H_2-biased response. Interestingly, Al(OH)_3_ adjuvanted CHIM_0265_1549 also was efficacious against H041 (WHO X) (Fig. 5). Analysis of IgG isotypes (Fig. 6) confirmed the T_H_1 and T_H_2 biases of the GLA-SE and Al(OH)_3_ adjuvanted vaccine responses, evidenced by a lower IgG1/IgG2a ratios in the GLA-SE group (median of 124.5 and 2.0 in the alum and GLA-SE groups, respectively; Fig. 6B). Total IgG responses of pooled antisera from each of the groups of mice used in experiments in Fig. 4 and 5 are shown in Fig. S4.

**Fig 7:**
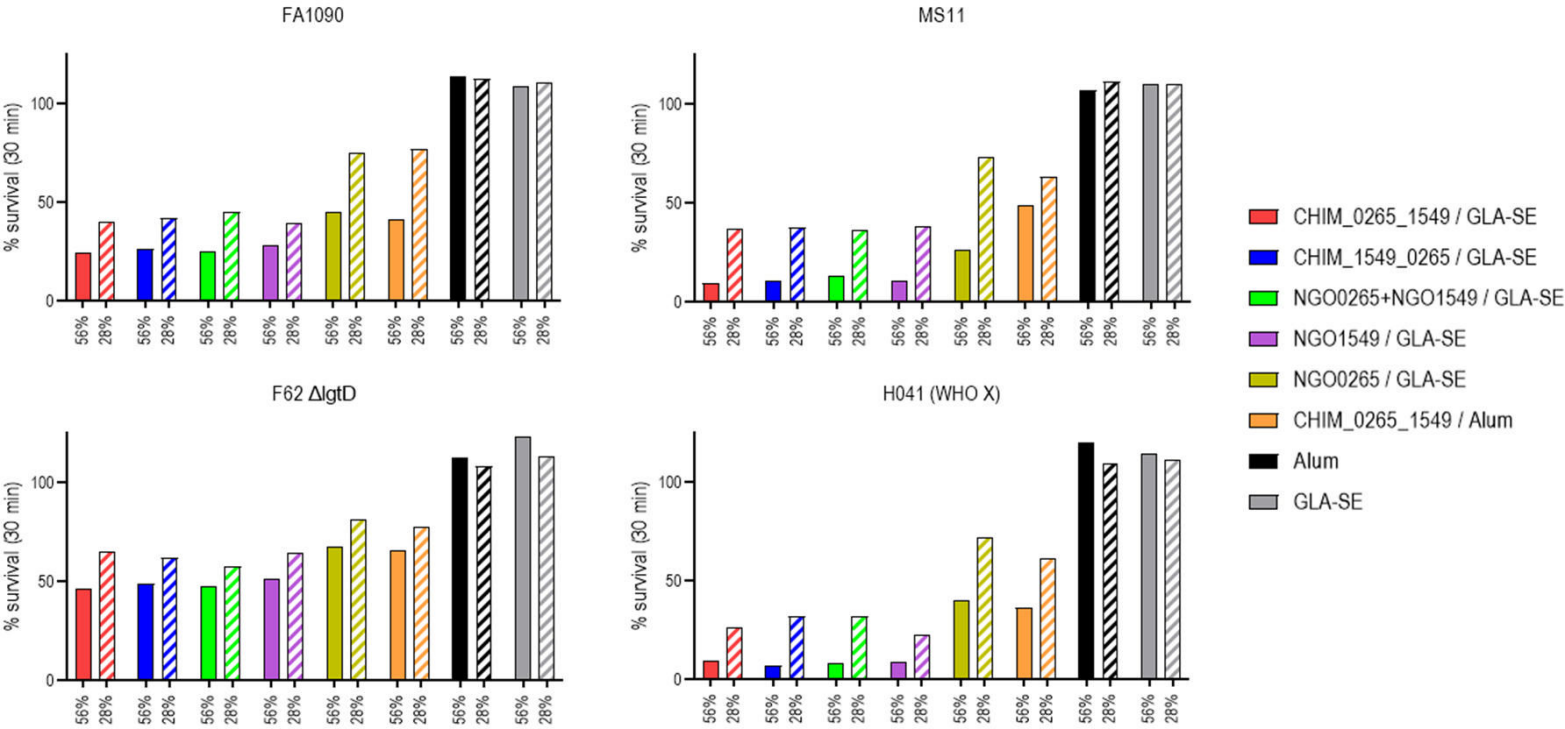
Complement-dependent bactericidal activity of IgG in immune sera elicited by NGO1549 (FtsN) and NGO0265 either as individual proteins, in combination (mixtures), or when expressed as chimeric proteins (NGO0265 is N-terminal in CHIM_0265_1549 and C-terminal in CHIM_1549_0265). Female BALB/c mice were immunized with the antigens and adjuvants (or adjuvant alone) as indicated in the legend. These sera were obtained from the same groups of mice immunized in Fig. 4 and 5, but which were not used in the challenge experiments. Immune sera in each group were pooled and depleted of mouse IgM by passage over anti-mouse IgM agarose. Pooled immune sera were added to each of the four test strains [FA1090, MS11, F62 ΔlgtD, and H041 (WHO X)] to a final concentration of 56% or 28% (solid and hatched bars, respectively), followed by human complement (11% for MS11 and 28% for the three other strains). Percent survival of bacteria at time 30 min relative to the start of the assay is indicated on the *Y*-axis.

We next determined the bactericidal activity of immune IgG elicited by each of the groups. Immune sera from mice in each group not used in challenge experiments in Fig. 4 and 5 were pooled and depleted of naturally occurring anti-*N*. *gonorrhoeae* bactericidal mouse IgM (16) by passage through anti-mouse IgM agarose. As shown in Fig. 7, the bactericidal activity of the sera correlated with efficacy *in vivo*; of the GLA-SE adjuvanted groups, those that received the chimeric proteins, a combination of NGO1549 and NGO0265, or with NGO1549 alone showed the greatest bactericidal activity, while the serum from NGO0265-immunized group showed intermediate bactericidal activity. Antisera from the CHIM_0265_1549 group adjuvanted with Al(OH)_3_ also showed intermediate bactericidal activity, although the decrease in AUC in this group was comparable with the GLA-SE adjuvanted groups with high *in vivo* efficacy and bactericidal activity.

### Efficacy of CHIM_0265_1549 *in vivo* is dependent on an intact terminal complement pathway

**Fig 8:**
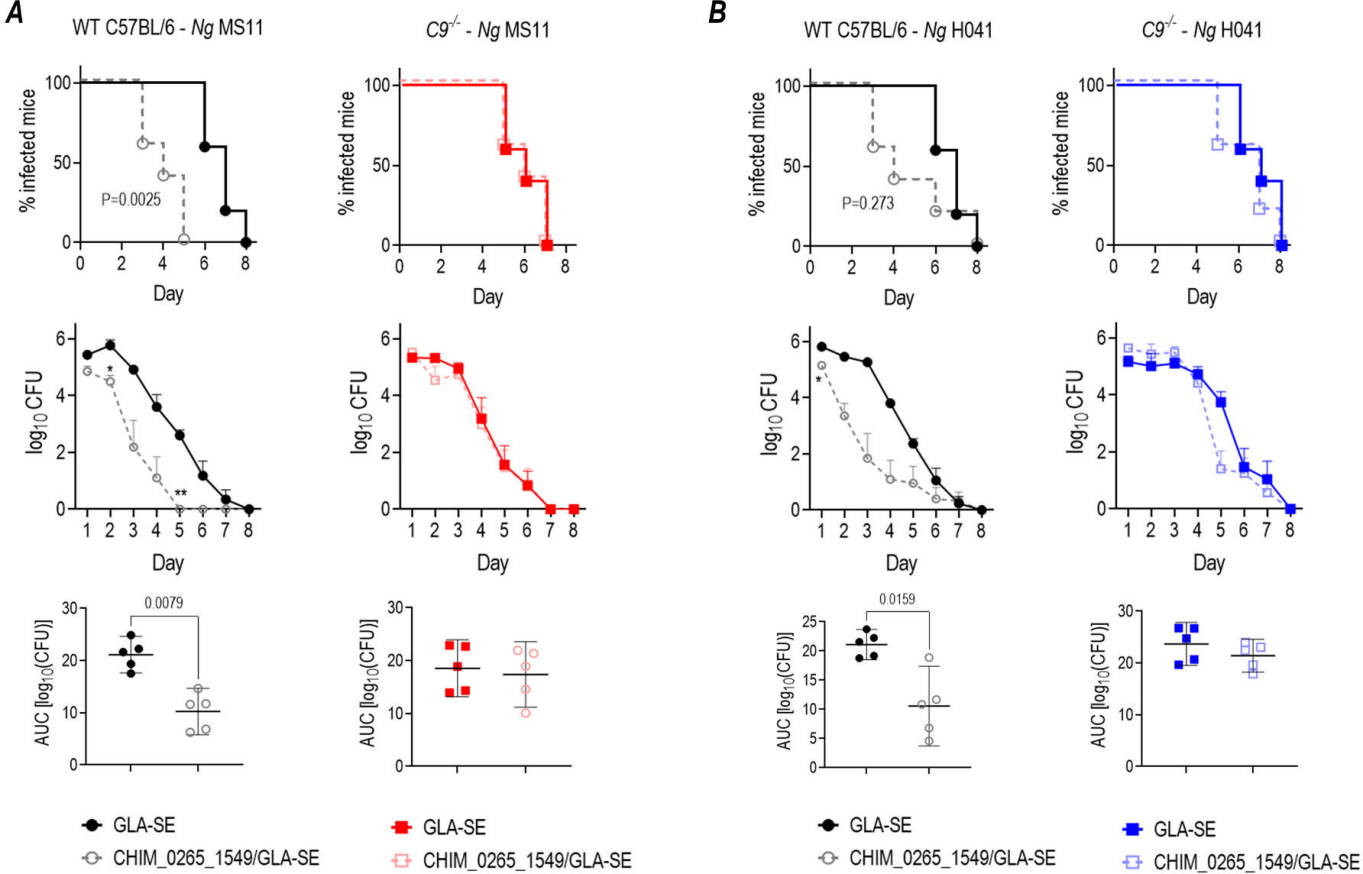
Efficacy of CHIM_0265_1549 requires an intact terminal complement pathway. WT C57BL/6 mice or complement *C9^−/−^* mice in a C57BL/6 background were immunized with CHIM_0265_1549 (25 µg/dose) formulated with GLA-SE (5 µg/dose), or GLA-SE alone intramuscularly at 0, 3, and 6 weeks. Two weeks post-dose 3, mice (*n* = 5 mice/group) were challenged intravaginally with either 2.6 × 10^7^ CFU *N*. *gonorrhoeae* MS11 (**A**) or 3.2 × 10^7^ CFU H041 (WHO X) (**B**) and vaginas were swabbed daily to enumerate CFUs. Top row: Kaplan–Meier curves for time to clearance. Comparisons across groups were made by Mantel-Cox log-rank test. Middle row: log_10_ CFU versus time, analyzed by two-way ANOVA (with Šídák’s multiple comparisons test). *P < 0.05 and **P < 0.01. Bottom row: AUC analysis (mean, ±95% confidence intervals). Comparisons across groups were made by Mann-Whitney’s non-parametric test.

To determine whether the activity of CHIM_0265_1549 required assembly of the membrane attack complex, we tested its efficacy in mice lacking complement C9 (*C9^−/−^* mice). Polymerization of C9 is the last step in the formation of the membrane attack complex pore. Wild-type C57BL/6 and *C9^−/−^* mice immunized with CHIM_0265_1549/GLA-SE were challenged with strain MS11 or H041 (WHO X) 2 weeks post-dose 3. While the vaccine was effective against both strains in wild-type mice, efficacy against both strains was abrogated in *C9^−/−^* mice, suggesting that an intact terminal complement pathway was required for the efficacy of CHIM_0265_1549 (Fig. 8).

### Breadth of bactericidal activity of antibodies raised against CHIM_0265_1549

**Fig 9:**
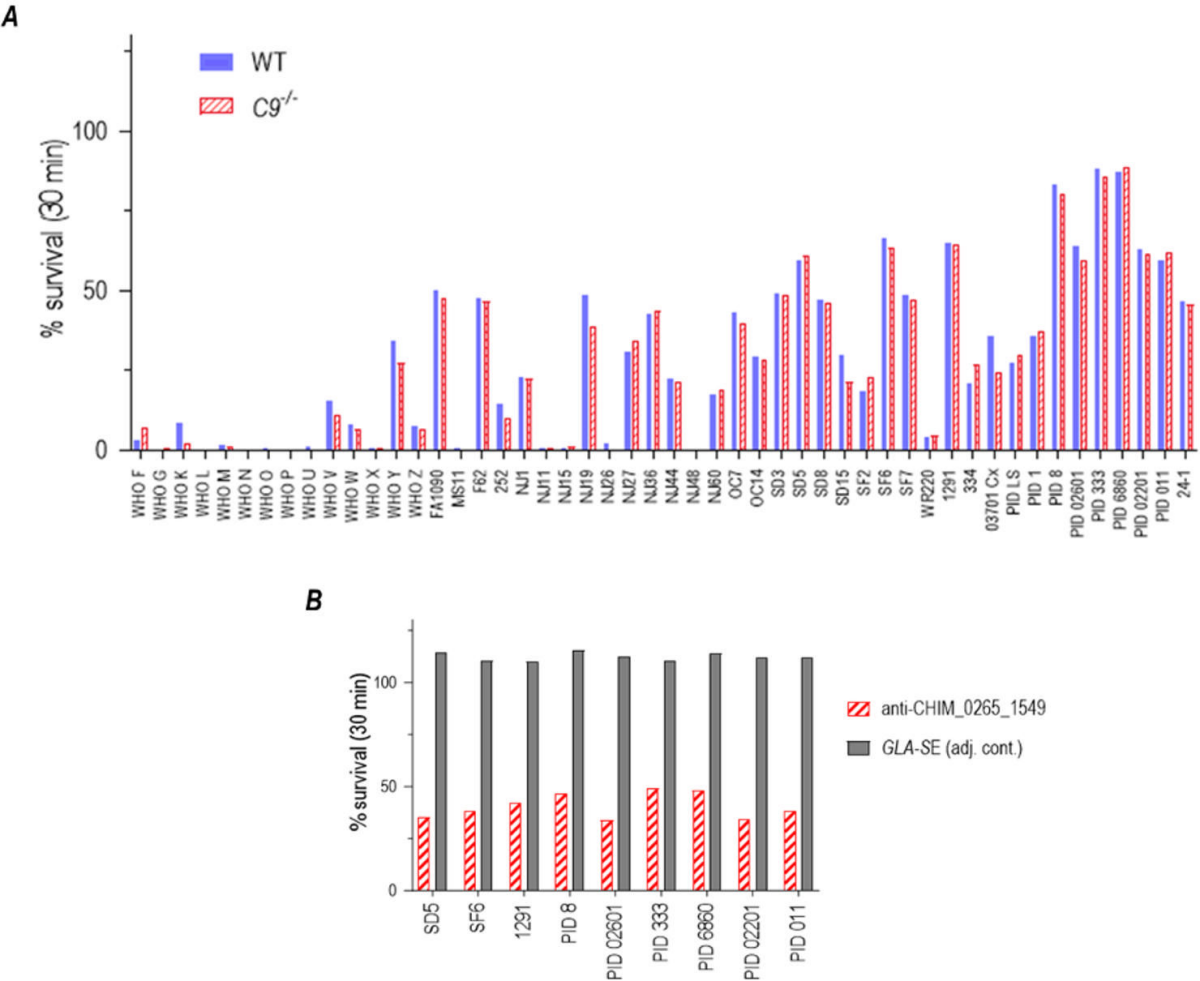
Bactericidal activity of anti-CHIM_0265_1549 antiserum elicited in C57BL/6 and *C9^−/−^* mice against a panel of 50 wild-type *N. gonorrhoeae* isolates. Antisera obtained from C57BL/6 and *C9^−/−^* mice immunized with CHIM_0265_1549 (Fig. 8) were pooled, depleted of mouse IgM, and tested for their ability to support killing of 50 wild-type gonococcal isolates in the presence of 20% human complement (IgG- and IgM-depleted normal human serum) as the complement source. (**A)** Bactericidal activity of antisera obtained from wild-type (solid blue bars) and *C9^−/−^* mice (red hatched bars) used at a dilution of 1:5 (final anti-serum concentration 20%). The gonococcal strains are listed on the *X*-axis. Percent survival (CFUs at 30 min relative to 0 min) is shown on the *Y*-axis. Immune serum or complement alone did not lead to the killing of any strains (>100% survival, data not shown). (**B)** Bactericidal activity of anti-CHIM_0265_1549 anti-serum obtained from *C9^−/−^* mice (red hatched bars) used at a 2:5 dilution (final anti-serum concentration 40%) against the nine gonococcal strains that showed greater than 50% survival in Fig. 9A. Percent survival using pooled sera obtained from mice given GLA-SE alone (adjuvant controls) is shown by the gray bars.

The requirement of C9 for the efficacy of CHIM_0265_1549 suggests that complement-dependent bactericidal activity is a mechanistic correlate of protection in mice. We next tested the ability of pooled anti-CHIM_0265_1549 sera at a final dilution of 1:5 to support bactericidal activity against a panel of 50 wild-type strains of *N. gonorrhoeae* (listed in Table S4). This assay also included sera pooled from *C9^−/−^* immunized mice to ensure that the lack of efficacy of the vaccine in these complement-deficient mice was not because of the absence of functional antibody. As shown in Fig. 9A, bactericidal activity (arbitrarily defined as ≤50% survival) was seen against 41/50 of sera from both WT and *C9^−/−^* animals that had been immunized (and not challenged). Both pools of sera displayed remarkably similar activity against all tested strains, suggesting that humoral immune responses in *C9^−/−^* mice were unimpaired. We next tested the ability of immune sera from *C9^−/−^* mice to support the killing of the nine isolates that survived >50% in 1:5 diluted (20%) serum using serum diluted 2:5 (final concentration 40%) and noted that all strains were killed >50% (Fig. 9B). Dose-dependent killing was evidenced by the observation that pooled WT and *C9^−/−^* antisera, when used at a 1:10 dilution, killed (<50% survival) only 13 and 17 of the 50 test strains, respectively (Fig. S5).

## DISCUSSION

This study has identified two promising gonococcal vaccine candidates using a machine learning algorithm, EDEN. We initially screened 26 gonococcal antigens for efficacy in the mouse vaginal colonization model of gonorrhea. While 8 of 11 groups showed efficacy against strain MS11 by at least one of two measures—time to clearance or area under curve—only the combination of NGO1549 and NGO0265 showed efficacy against strain H041 (WHO X). While combining two to three antigens into groups permitted evaluation of all antigens in a single experiment, a limitation of pooling candidates is the possibility that subversive immune responses, such as blocking antibodies (24–27), elicited by one antigen may mask protection by a potentially protective antigen. Of note, Rmp, a known target for gonococcal blocking antibodies (24), did not appear in the EDEN rank as a potential protective immunogen. Coupled with the broad bactericidal activity of sera obtained from this group, NGO1549 and NGO0265 were selected for further studies as lead candidates. NGO1549 is the gonococcal homolog of the cell division protein FtsN and is essential for neisserial viability (28). FtsN belongs to the neisserial cell division interactome. Using a bacterial two-hybrid system, Zou et al. showed that FtsN interacted with FtsK and FtsA (29). NGO0265 contains an FtsN motif and shares 28% identity with NGO1549. While not essential for viability, a previous study showed that deleting NGO0265 caused (i) changes in cell morphology (rough colony with growth as clusters of four indicative of a cell separation defect, hence the genotype pneumonic “tetrapac”), (ii) complete loss of natural competence, (iii) decreased murein hydrolase activity, and (iv) diminished epithelial cell invasion (30). Genes encoding both proteins are found in >99.99% of 12,213 unfinished *Ng* genome assemblies (PubMLST). A proteomic analysis carried out by Zielke et al. reported NGO1549 as one of 305 ubiquitously expressed cell envelope proteins (31).

Although the exact cellular location of NGO0265 and FtsN as predicted by pSORTb (Table S2) remains unclear, IgG in antiserum raised against these two proteins bound to live bacteria by flow cytometry (Fig. S3B). Antisera against these proteins were also bactericidal, providing further evidence that at least some epitopes of these proteins are accessible to IgG. It is possible that FtsN and NGO0265 function as “moonlighting proteins”; such microbial proteins, while not predicted to be surface expressed (or exposed), may be present there ectopically and perform additional functions such as adhesion and scavenging complement inhibitors (32–38). Another possibility is autolysis of gonococci, where released antigens could bind to the surface of intact bacteria. Reasons for the relatively lower bactericidal activity of anti-CHIM_0265_1549 sera against the nine strains shown in Fig. 9B are not clear, but less effective complement activation because of lower antibody binding to live bacteria is a possibility that merits further investigation.

A hurdle in the development of gonococcal vaccines has been the lack of a correlate of protection. Although 4CMenB and MeNZB vaccines have been associated with lower rates of gonorrhea compared to control groups (5–8), the mechanism of protection has not been elucidated. Immunization with 4CMenB elicits antibodies that cross-react with *N. gonorrhoeae* (39); however, post-immune sera do not show significant increases in bactericidal activity compared to pre-immune sera (40, 41). An experimental gonococcal vaccine comprising OMVs and microsphere-encapsulated interleukin-12 induces T_H_1-driven immunity, with circulating and genital antibodies to *N. gonorrhoeae*, after intravaginal or intranasal administration in female mice (10, 11, 18). This candidate vaccine relies on antibody production, evidenced by the loss of efficacy in µMT mice (11). It remains unclear whether complement is required for the function of antibodies elicited by this vaccine candidate. Antisera raised in mice against MetQ and NHBA have both shown bactericidal activity against gonococci (12, 13). A separate study showed that MetQ adjuvanted with CpG reduced gonococcal colonization of mouse vaginas, but the mechanism of vaccine action was not defined (14). Mice immunized with MtrE or MtrE loop 2 configured as an oligomer by fusion with the oligomerization domain of C4b-binding protein elicited T_H_1-biased bactericidal antibodies and were protected against vaginal gonococcal infection (42, 43). Whether the efficacy of the MrtE-based vaccine in mice required complement-dependent killing remains unclear. Matthias et al. showed the activity of meningococcal OMV-based vaccines against *N. gonorrhoeae* in the mouse vaginal colonization model, but immune sera did not support complement-dependent bactericidal activity against gonococci grown in CMP-Neu5Ac-containing media to sialylate LOS, as occurs *in vivo* (15). In this study, we noted a positive correlation between the bactericidal activity of immune sera and the percent reduction in bacterial burden in female mice, which suggested a role for complement-dependent killing for vaccine efficacy. Consistent with this hypothesis, the lack of CHIM_0265_1549 efficacy in *C9^−/−^* mice, which have intact opsonophagocytosis and adaptive immune responses (44), strongly suggested that killing by the membrane attack complex of complement, and not phagocytic killing, was necessary for vaccine activity. The neonatal Fc receptor (FcRn) present in female genital tract epithelial cells mediates the transport of IgG into the genital secretions (45). Furthermore, complement components are synthesized by primary cervical epithelial cells (46) and cervical secretions contain hemolytically active complement (47). Thus, the milieu of the female genital tract is capable of supporting complement-dependent killing of gonococci by vaccines that elicit systemic bactericidal IgG responses. We previously reported terminal complement-dependent efficacy of a peptide vaccine candidate that mimics a conserved gonococcal LOS epitope (44). Considering the numerous mechanisms that gonococci have evolved to evade killing by neutrophils (48–52), it is not surprising that this organism subverts antibody-mediated opsonophagocytosis. We acknowledge that our observations may be specific to the female mouse genital tract; further work is necessary to establish the efficacy and mechanisms of action of vaccines in different anatomic niches in men and women.

A vaccine containing a combination of antigens—for example, the chimeras of NGO0265 and NGO1549—may be advantageous for the following reasons. Antibodies directed against multiple epitopes or antigens could potentially synergize because increasing antibody density on the bacterial surface enhances engagement of the C1 complex and increases the efficiency of complement-dependent killing (53, 54). The barrier for the emergence of vaccine escape mutants would be considerably higher against vaccine combinations because it would require downregulation or turning off expression, or mutation of epitopes targeted by bactericidal antibodies in more than one target antigen. One of the candidate antigens, FtsN (NGO1549), is essential for bacterial viability, thus expression cannot be turned off.

In conclusion, the machine learning platform EDEN has identified two promising gonococcal antigens, which when used in combination as a chimeric have elicited functional bactericidal antibodies *in vitro* and have shown efficacy in a pre-clinical animal model. Our findings highlight EDEN’s ability to predict protein-specific antibody-mediated protection and the utility of the EDEN AI platform to rapidly identify novel vaccine candidates that have not previously been considered using more traditional means.

## MATERIALS AND METHODS

### Efficacy discriminative educated network

EDEN is an AI-driven platform trained to identify novel, protective antigens for use in vaccines against pathogenic bacteria. The core of EDEN is a proprietary machine learning ensemble of AI models used to interpret immunological-relevant information in relation to bacterial antigens that confer protection when administered as vaccines. EDEN has been trained on EVAXION’s curated data set derived from publicly available data (publications and patents) describing protective and non-protective antigens tested in clinical and pre-clinical settings. The input to these AI models is a feature vector transformation of the full-length protein amino acid sequence, from which several global and sequence-resolved properties are extracted. These structural and functional features have been selected for their relevance in protein chemistry and structure, immunology, and their ability to guide the network in discriminating protective versus non-protective antigens.

The application of EDEN consists of four key steps: (i) collection of clinically relevant pathogen proteomes, (ii) proteome-wide processing with EDEN, (iii) selection and design of predicted protein antigens, and (iv) testing in preclinical *in vivo* and *in vitro* models. In step 1, to identify broadly protective bacterial vaccine antigens, proteomes from clinically relevant strains are collected as input for EDEN. The protein-coding regions of such strains are translated into amino acid sequences. Step 2 aims to discriminate protective from non-protective antigens by identifying unique features or combinations of features associated with protection. An example is the antigen presentation machinery feature along with antibody recognition features to predict protective antigens. EDEN ranks all proteins in the pathogen’s proteome from 0 to 1, based on their probability of eliciting a protective immune response. The EDEN score, together with the calculated sequence conservation across a selected pool of 106 NCBI scaffold strain proteomes (strains listed in Table S5), was used to identify the centroid sequence (i.e., the sequence least divergent from all strains). The centroid sequence was used to construct the recombinant antigens in an attempt to achieve the best possible strain coverage. Step 4 involves experimental verification of the candidacy of the antigen.

The CPS was derived as a measure of the protein’s ability to protect across protein homologs and broadly across strains within a species. CPS was calculated by the summation of the products of the strain-specific B-cell antigen prediction score with the strain-specific conservation value %ID, defined by the equation


$$cross\;protection\;score\;\left(CPS\right)=\sum^{strains}\left(eden\_pred\_score\_hom*\%ID\_prim\_vs\_hom\right)$$

where eden_pred_score_hom is the strain-specific predicted B-cell antigen protection score and %ID_prim_vs_hom is the percent identity in pairwise sequence alignment between selected primary strain protein sequence and homolog strain protein sequence.

### Bacterial strains

FA1090 (55), MS11 (56), F62 ΔlgtD (mutant in F62 incapable of adding GalNAc to lacto-N-neotetraose extension from heptose I [HepI] [57]), and H041 (also called WHO X) (58) have been described previously. H041 was rendered streptomycin-resistant for mouse infection experiments as described previously (59). *ngo0265* was deleted in WHO X (H041) by replacing the open-reading frame with a kanamycin-resistance marker. Table S4 lists the PorB serotype and genetic characteristics of the 50 strains used to evaluate the bactericidal activity of anti-CHIM_0265_1549 antiserum.

### Recombinant proteins and preparation of immunogens

Recombinant proteins were expressed in *E. coli* strain BL21 (DE3) and purified at either GenScript or Creative Biomart. Unless otherwise specified, the proteins were His-tagged to facilitate purification. Descriptions of the proteins and purity as assessed by Coomassie blue staining of SDS-PAGE are provided in Table S1. Soluble antigens were formulated in PBS and were mixed with GLA-SE with gentle pipetting to yield a suspension where 100 µL contained 15 µg of each individual protein (or 25 µg of a chimeric protein used) plus 5 µg of glucopyranosyl lipid A in a stable squalene-based oil-in-water emulsion adjuvant (GLA-SE). Insoluble antigens that were stored in urea (NGO0275, NGO0225, NGO1495, NGO2093, NGO1392, NGO1585, NGO2109, NGO1801, NGO0952, and NGO1715) were first mixed with Alhydrogel 2% [Al(OH)_3_] at a ratio of 100 µL of Alhydrogel to 125 µg of protein with end-over-end rotation for 1 h. A volume of NaCl (0.9%) twice the original reaction mixture was added, followed by end-over-end mixing for another 20 min. This step was repeated twice to reduce the final concentration of urea in the mixture to 1 M. The protein-alum complex was washed twice in 0.9% NaCl by centrifugation at 1,000 *g* for 2 min. The pellet was then resuspended in an appropriate volume of 0.9% NaCl prior to mixing with GLA-SE. The insoluble proteins were used at 30 µg per dose.

### ELISA

For determining total IgG responses against recombinant proteins (Fig. S1 and S4), Immulon 2 U-bottomed plates were coated with recombinant antigens (1 µg/mL in PBS) or with bacterial lysates (bacterial pellets frozen at −20°C, which were subsequently thawed) for 3 h at 37°C and then placed at 4°C for 15 h. Plates were washed/blocked with PBS/Tween 20 for 1 h at 22°C and incubated with serial dilutions of antisera for 1 h at 37°C. Bound IgG was detected with anti-mouse IgG conjugated to alkaline phosphatase and para-nitrophenyl phosphate substrate.

IgG subclass determination in immune sera was determined as follows. MaxiSorp microtiter 96-well plates (Thermo Fisher Scientific, Cat. No. 442404) were coated overnight at 4°C with 1 µg/mL recombinant vaccine protein in PBS (100 µL/well). Plates were then washed twice with 200 µL/well PBS/0.05% Tween 20 and blocked with PBS containing 10% fetal bovine serum (FBS) for 30 min at 22°C. Sera, in twofold dilution series starting at 1:200 in PBS, were added at 100 µL/well and incubated for 2 h at RT on an orbital shaker. Microtiter plates were then washed three times with PBS containing 0.05% Tween20 and then incubated with 100 µL/well horseradish peroxidase (HRP)-conjugated secondary antibody (goat anti-mouse IgG1, goat anti-mouse IgG2a, goat anti-mouse IgG2b, or goat anti-mouse IgG3) for 1 h at RT on an orbital shaker. Following three washes with PBS/0.05% Tween 20, wells were incubated TMB-Ease (Kementec, Cat No. 5320) for 35 min at RT in the dark. After 35 min, the reaction was stopped with 100 µL/well 0.2 M H_2_SO_4_, and optical density at 450 nm was recorded.

### Serum bactericidal assay

Serum bactericidal assays were performed using a modification of previously described methods (16). The final volume of the reaction mixture was reduced to 90 µL to reduce the amount of antiserum used. The final concentration of human complement in reactions was 28% for all strains, except MS11 (11% complement) because of its greater susceptibility to complement. Survival at 30 min relative to colony forming unit (CFU) at 0 min was expressed as a percentage.

### Mouse infection experiments

Female wild-type BALB/c mice (The Jackson Laboratory) in the diestrus phase of the estrous cycle were started on treatment (that day) with 0.5 mg Premarin (Pfizer) in 200 µL water given subcutaneously on each of 3 days; −2, 0, and +2 days (before, the day of, and after inoculation) to prolong the estrus phase of the cycle and promote susceptibility to *N. gonorrhoeae* infection (60). Premarin is a mixture of sodium estrone sulfate and sodium equilin sulfate and as concomitant components, sodium sulfate conjugates of 17α-dihydroequilin, 17α-estradiol, and 17β-dihydroequilin. Antibiotics (vancomycin, colistin, neomycin, trimethoprim, and streptomycin) ineffective against *N. gonorrhoeae* were used to reduce competitive microflora (60). Mice were challenged intravaginally with the indicated inoculum of *N. gonorrhoeae* as previously described (61). Infection was monitored daily through vaginal swabbing and bacterial enumeration (CFUs).

### Statistical analyses

For challenge experiments in mice, three characteristics of the data were measured (59, 62): time to clearance, longitudinal trends in mean log_10_ CFU, and the cumulative CFU as AUC. Median time to clearance was estimated using Kaplan–Meier survival curves; times to clearance were compared between groups using the Mantel-Cox log-rank test. Significance was set using Bonferroni’s correction when more than two groups were compared and is indicated for each experiment. The mean AUC (log_10_ CFU) was computed for each mouse to estimate the bacterial burden over time (cumulative infection); the means under the curves were compared between groups using Kruskal-Wallis and Dunn’s *post hoc* test.

Linear regression was performed to assess the relationship between the percent reduction in AUC and *in vitro* bactericidal activity. *Z*-score (or the standard score) was calculated using the formula $Z=\frac{x-\mu }{\sigma }$ , where *x* is the observed value, *µ* is the mean, and *σ* is the standard deviation of the group. Tables S2 and S3 list the AUCs, bactericidal assay results, and *Z*-scores.

A non-linear regression analysis was used to calculate the EC50 (also called the half-maximum titers) for IgG subclass analysis, which is the serum dilution estimated to provide an OD reading halfway between the bottom and the highest (saturated) readings.

## SUPPLEMENTAL MATERIAL

Fig. S1 mbio.02500-23-s0001.pdf IgG responses against vaccine antigens.

Fig. S2 mbio.02500-23-s0002.pdf Bactericidal activity of vaccine antisera.

Fig. S3 mbio.02500-23-s0003.pdf Expression levels of NGO0265 and NGO1549 by five strains of *N. gonorrhoeae*.

Fig. S4 mbio.02500-23-s0004.pdf IgG responses in mice immunized with NGO0265 and NGO1549, either alone or in combination (mixtures) or when configured as chimeric proteins.

Fig. S5 mbio.02500-23-s0005.pdf Bactericidal activity of anti-CHIM_0265_1549 antiserum (1:10 dilution) elicited in C57BL/6 and C9^-/-^ mice against a panel of 50 *N. gonorrhoeae* isolates.

Table S1 mbio.02500-23-s0006.xlsx Description of antigens used for immunizations in Fig. 1.

Table S2 mbio.02500-23-s0007.pdf Prediction of localization of vaccine antigens by PSORTb.

Table S3 mbio.02500-23-s0008.pdf EDEN prediction scores and derivation of AUC and bactericidal Z-scores for *N. gonorrhoeae* MS11 and H041 (WHO X).

Table S4 mbio.02500-23-s0009.pdf Characteristics of the 50 strains of *N. gonorrhoeae* used in Fig. 9 and Fig. S5.

Table S5 mbio.02500-23-s0010.pdf Strains used to identify centroid sequences of antigens.

ASM does not own the copyrights to Supplemental Material that may be linked to, or accessed through, an article. The authors have granted ASM a non-exclusive, world-wide license to publish the Supplemental Material files. Please contact the corresponding author directly for reuse.
