# Supplementary material for: Preclinical efficacy of a cell division protein candidate gonococcal vaccine identified by artificial intelligence
Source: mBio. 2023 Oct 31;14(6):e02500-23. doi: 10.1128/mbio.02500-23 (PMC10746169; doi:10.1128/mbio.02500-23)
Supplement: Fig. S1 — IgG responses against vaccine antigens. [file mbio.02500-23-s0001.pdf]

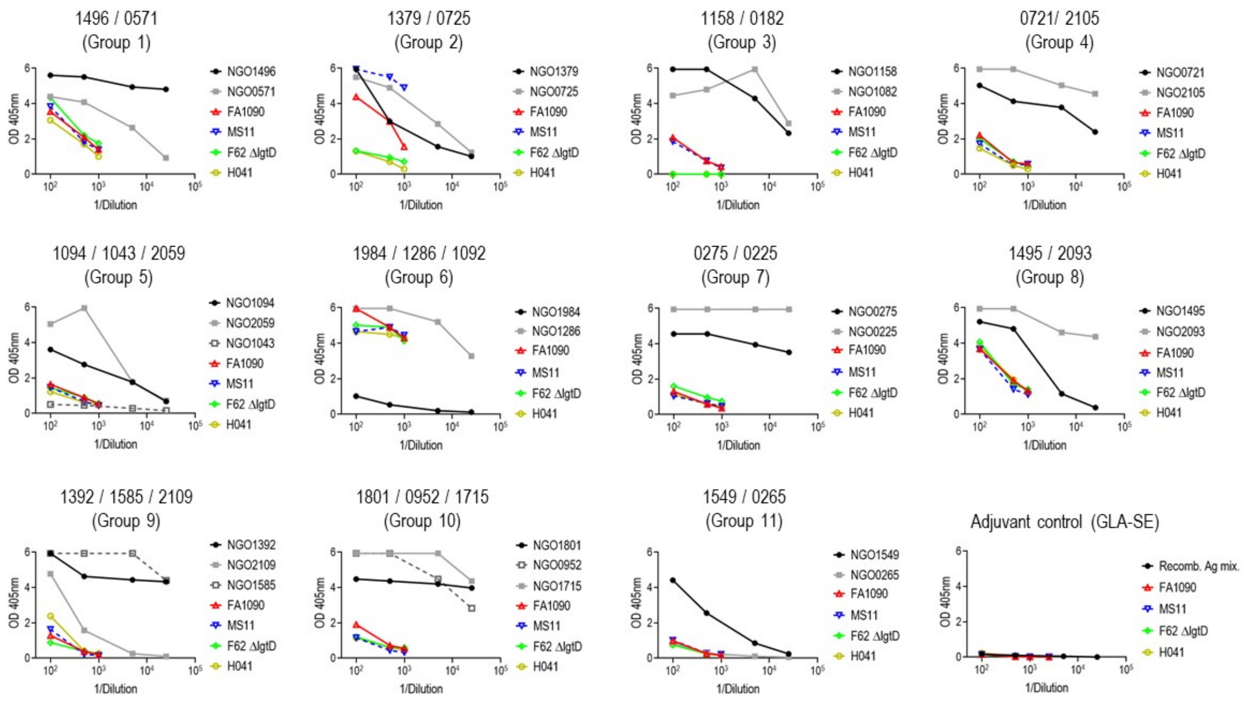

Figure S1. IgG responses against purified protein antigens and bacterial lysates in sera collected from 11 groups of mice immunized with the 26 antigens, as shown in Figure 1. Wells were coated with 20 µg/mL of each purified antigen. Immune sera collected from 5 mice not used in protection experiments were pooled and IgG levels against the respective purified proteins and whole cell lysates from strains FA1090, MS11, F62 ΔlgtD and H041 (WHO X) were measured. Serum dilutions of 1/100, 1/500, 1/5,000 and1/25,000 against purified proteins and at 1/100, 1/500 and 1/1,000 against bacterial lysates were used (indicated on the X-axis). The Y-axis indicates OD_{405nm} readings at 30 min.
