# Supplementary material for: Preclinical efficacy of a cell division protein candidate gonococcal vaccine identified by artificial intelligence
Source: mBio. 2023 Oct 31;14(6):e02500-23. doi: 10.1128/mbio.02500-23 (PMC10746169; doi:10.1128/mbio.02500-23)
Supplement: Fig. S2 — Bactericidal activity of vaccine antisera. [file mbio.02500-23-s0002.pdf]

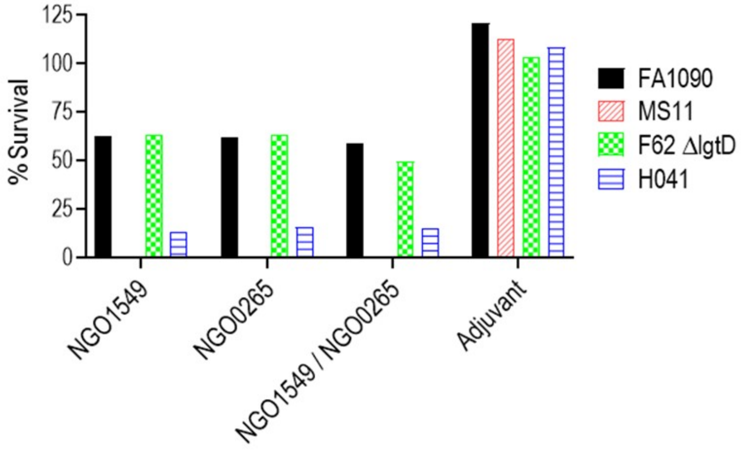

Figure S2. Bactericidal activity against *N. gonorrhoeae* of sera elicited by NGO1549 (FtsN) and NGO0265 adjuvanted with GLA-SE. Immune sera from mice not challenged with *N. gonorrhoeae* in the experiment in **Fig. 3** were pooled and depleted of mouse IgM. IgM-depleted mouse immune sera or serum from mice immunized with adjuvant alone (adjuvant control serum (50 µL in a final reaction volume of 90 µL; final concentration 56%)) was incubated with strains FA1090, MS11, F62 ΔlgtD and H041 (WHO X) together with human complement (IgG and IgM depleted normal human serum; final concentration 28% for all strains, except MS11, where a final concentration of 11% was used). Percent survival at 30 min relative to 0 min is indicated on the Y-axis. The data represent one single experiment.
