# Supplementary material for: Preclinical efficacy of a cell division protein candidate gonococcal vaccine identified by artificial intelligence
Source: mBio. 2023 Oct 31;14(6):e02500-23. doi: 10.1128/mbio.02500-23 (PMC10746169; doi:10.1128/mbio.02500-23)
Supplement: Fig. S3 — Expression levels of NGO0265 and NGO1549 by five strains of N. gonorrhoeae. [file mbio.02500-23-s0003.pdf]

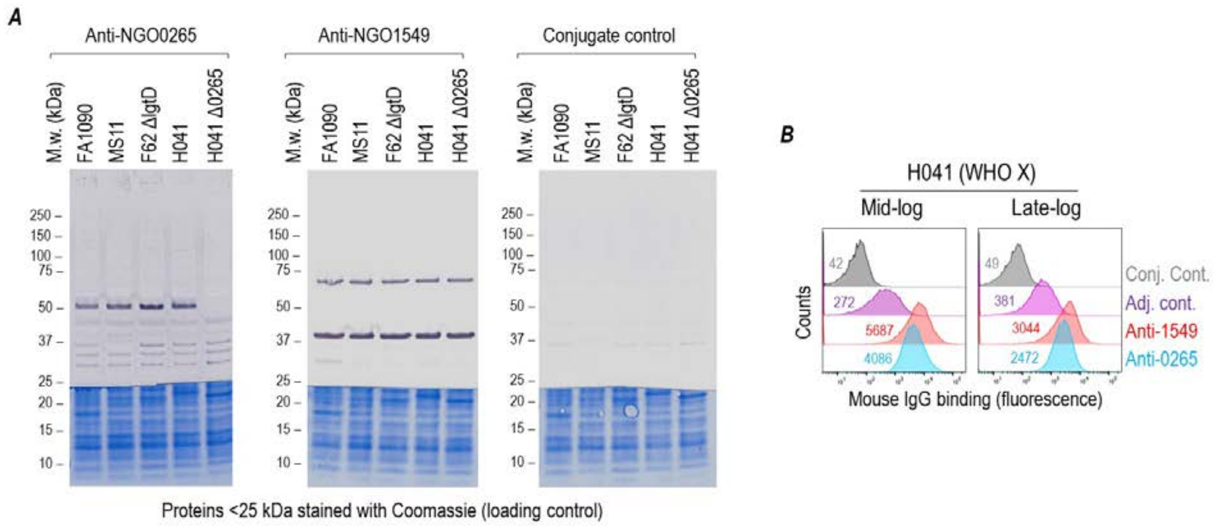

Figure S3. Expression levels of NGO0265 and NGO1549 by five strains of *N. gonorrhoeae* and binding of IgG in anti-NGO1549 (FtsN) and anti-NGO0265 antisera to live H041 (WHO X) bacteria. ***A***. Lysates of *N. gonorrhoeae* strains FA1090, MS11, F62 ΔlgtD, H041 (WHO X) and H041 Δ0265 (NGO1549 is essential for gonococcal viability hence could not be deleted) were separated on a 4-12% Bis-Tris gel using MES running buffer and proteins were transferred to a PVDF membrane by wester blotting. Membranes were cut horizontally at the 25 kDa marker and the lower portion was stained with Coomassie blue that served as a loading control. The upper portion was probed with either anti-NGO0265 (left blot) or anti-NGO1549 (middle blot) and bound antibodies detected with anti-mouse IgG alkaline phosphatase and developed with Pierce™ diethanolamine buffer (Cat. No. 34064). The blot on the right was treated with anti-mouse IgG alkaline phosphatase (conjugate control). ***B***. Anti-NGO1549 and anti-NGO0265 antisera were incubated with WHO X (H041) grown either to the mid-log or late-log phases and bacteria-bound IgG was detected by flow cytometry using anti-mouse IgG FITC (Sigma; final dilution 1:100) as the fluorescent conjugate. The conjugate control (labeled 'Conj. Cont.') were bacteria incubated with the fluorescent conjugate alone, and the adjuvant control (labeled 'Adj. cont.') were bacteria incubated with antiserum from GLA-SE (adjuvant) immunized mice. All antisera were used at a concentration of 20%.
