# Supplementary material for: Preclinical efficacy of a cell division protein candidate gonococcal vaccine identified by artificial intelligence
Source: mBio. 2023 Oct 31;14(6):e02500-23. doi: 10.1128/mbio.02500-23 (PMC10746169; doi:10.1128/mbio.02500-23)
Supplement: Fig. S4 — IgG responses in mice immunized with NGO0265 and NGO1549, either alone or in combination (mixtures) or when configured as chimeric proteins. [file mbio.02500-23-s0004.pdf]

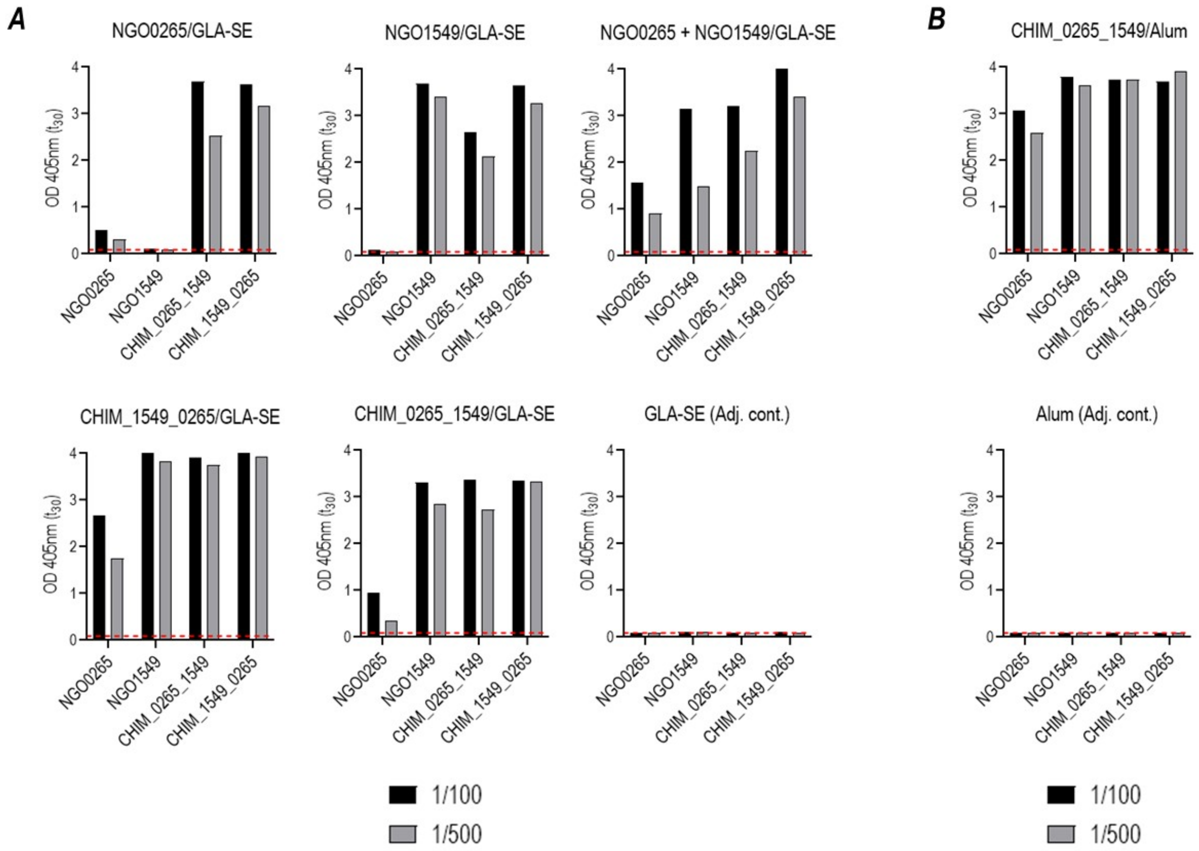

Figure S4. IgG responses in mice immunized with NGO0265 and NGO1549, either alone or in combination (mixtures) or when configured as chimeric proteins. Sera obtained 2 weeks post-dose 3 from 5 mice in each group in the experiments shown in Fig. 4 and Fig. 5 were pooled and diluted 1/100 or 1/500 and tested for reactivity of IgG against NGO0265, NGO1549, CHIM_0265_1549 and CHIM_1549_0265 immobilized onto microtiter wells. The title of each graph indicates the vaccine immunogen (or adjuvant control), the X-axis indicates the protein immobilized onto microtiter wells and the Y-axis shows the OD_{405nm} reading at 30 min in a single experiment. The horizonal red dashed line indicates the conjugate control (background) readings.
