# Supplementary material for: Preclinical efficacy of a cell division protein candidate gonococcal vaccine identified by artificial intelligence
Source: mBio. 2023 Oct 31;14(6):e02500-23. doi: 10.1128/mbio.02500-23 (PMC10746169; doi:10.1128/mbio.02500-23)
Supplement: Fig. S5 — Bactericidal activity of anti-CHIM_0265_1549 antiserum (1:10 dilution) elicited in C57BL/6 and C9-/- mice against a panel of 50 N. gonorrhoeae isolates. [file mbio.02500-23-s0005.pdf]

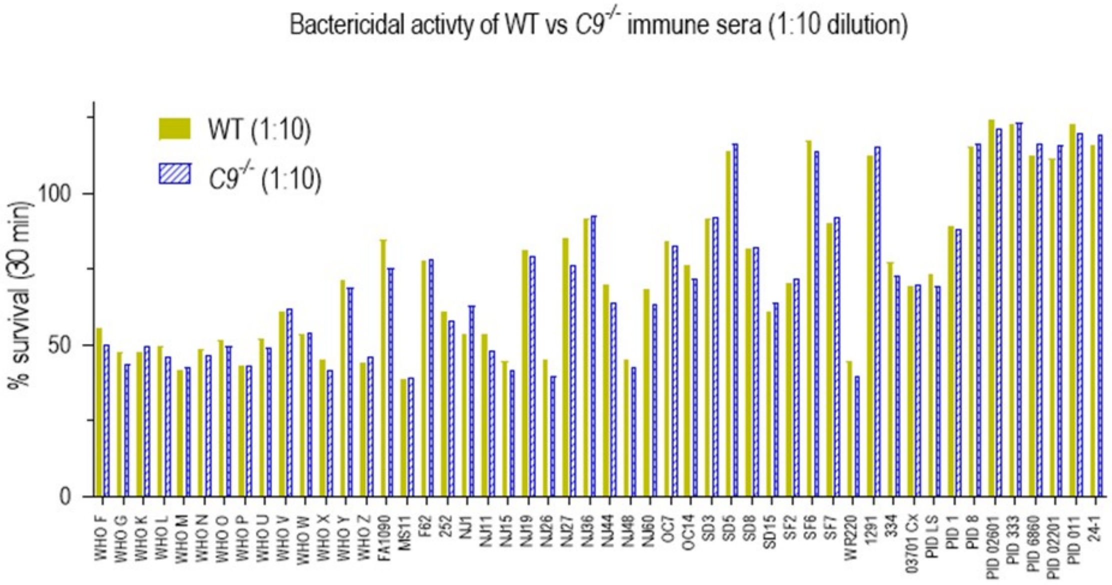

Figure S5. Bactericidal activity of anti-CHIM_0265_1549 antiserum elicited in C57BL/6 and *C9*$^{-/-}$ mice against a panel of 50 *N. gonorrhoeae* isolates. Immune sera obtained from C57BL/6 and *C9*$^{-/-}$ mice immunized with CHIM_0265_1549 (Fig. 8) were pooled, depleted of mouse IgM and tested at a dilution of 1:10 for bactericidal activity in the presence of 20% human complement (IgG and IgM depleted normal human serum) against the 50 strains listed on the X-axis. Percent survival (CFUs at 30 min relative to 0 min) is shown on the Y-axis. Immune serum or complement alone did not kill any strain (>100% survival, data not shown).
