# Supplementary material for: Preclinical efficacy of a cell division protein candidate gonococcal vaccine identified by artificial intelligence
Source: mBio. 2023 Oct 31;14(6):e02500-23. doi: 10.1128/mbio.02500-23 (PMC10746169; doi:10.1128/mbio.02500-23)
Supplement: Table S2 — Prediction of localization of vaccine antigens by PSORTb. [file mbio.02500-23-s0007.pdf]

Table S2. Prediction of localization of vaccine antigens by PSORTb

Group No	Protein ID	PSORTb version 3.0.3						
		Final Localization	Scores					
			Final	Cytoplasmic	Cytoplasmic Membrane	Periplasmic	Outer Membrane	Extracellular
1	NGO1496	Extracellular	9.64	0.01	0.01	0.11	0.23	9.64
	NGO0571	OuterMembrane	9.52	0.00	0.01	0.09	9.52	0.38
2	NGO1379	Unknown	2.00	2.00	2.00	2.00	2.00	2.00
	NGO0725	Unknown	2.00	2.00	2.00	2.00	2.00	2.00
3	NGO1158	Unknown	2.50	0.00	2.50	2.50	2.50	2.50
	NGO0182	CytoplasmicMembrane	7.88	2.11	7.88	0.00	0.00	0.00
4	NGO0721	Cytoplasmic	8.96	8.96	0.51	0.26	0.01	0.26
	NGO2105	Unknown (This protein may have multiple localization sites)	5.87	0.00	0.00	0.00	5.87	4.13
5	NGO1094	Cytoplasmic	8.96	8.96	0.51	0.26	0.01	0.26
	NGO1043	Periplasmic	9.84	0.00	0.01	9.84	0.01	0.14
	NGO2059	Cytoplasmic	9.26	9.26	0.24	0.48	0.01	0.01
6	NGO1984	Unknown	2.00	2.00	2.00	2.00	2.00	2.00
	NGO1286	Cytoplasmic	9.94	9.94	0.00	0.04	0.00	0.00
	NGO1092	OuterMembrane	9.52	0.00	0.01	0.09	9.52	0.38
7	NGO0275	Extracellular	9.46	0.00	0.00	0.00	0.54	9.46
	NGO0225	Unknown (This protein may have multiple localization sites)	4.90	0.00	4.90	2.50	0.10	2.50
8	NGO1495	OuterMembrane	10.00	0.00	0.00	0.00	10.00	0.00
	NGO2093	OuterMembrane	10.00	0.00	0.00	0.00	10.00	0.00
9	NGO1392	Unknown	2.50	0.00	2.50	2.50	2.50	2.50
	NGO1585	Periplasmic	9.84	0.00	0.01	9.84	0.01	0.14
	NGO2109	OuterMembrane	10.00	0.00	0.00	0.00	10.00	0.00
10	NGO1801	OuterMembrane	10.00	0.00	0.00	0.00	10.00	0.00
	NGO0952	OuterMembrane	10.00	0.00	0.00	0.00	10.00	0.00
	NGO1715	OuterMembrane	10.00	0.00	0.00	0.00	10.00	0.00
11	NGO1549	Unknown	2.00	2.00	2.00	2.00	2.00	2.00
	NGO0265	Unknown	2.50	0.00	2.50	2.50	2.50	2.50
