# Supplementary material for: Preclinical efficacy of a cell division protein candidate gonococcal vaccine identified by artificial intelligence
Source: mBio. 2023 Oct 31;14(6):e02500-23. doi: 10.1128/mbio.02500-23 (PMC10746169; doi:10.1128/mbio.02500-23)
Supplement: Table S3 — EDEN prediction scores and derivation of AUC and bactericidal Z-scores for N. gonorrhoeae MS11 and H041 (WHO X). [file mbio.02500-23-s0008.pdf]

Table S3A. EDEN prediction scores, and derivation of AUC and bactericidal Z-scores for *N. gonorrhoeae* MS11

Challenge Group	Recombinant Centroid Construct	Challenge strain: MS11								
		EDEN prediction score (of challenge homolog target proteins)			PROTECTION (*in vivo*)				PROTECTION (*in vitro*)	
	**Protein ID** "c" = centroid homolog is not a FA1090 strain protein	**Locus ID** (challenge homolog target protein)	**EDEN_pred_score_homolog**	**Group Mean (EDEN_pred_score_homolog)**	**AUC (log10 CFU)** [P-value, mann-whitney-test]	**Z-score normalized** (p-value)	**AUC (log10 CFU)** [% Median Reduction, Test group vs Ctrl group]	**Z-score normalized** (% median reduction)	**% bacterial killing**	**Z-score normalized**
1	cNGO1496 (1-693 AA)	NGFG_RS10870	0.9849	0.9840	0.0079	-0.5581	37.2%	1.4336	100.0%	1.065
	cNGO0571 (21-598 AA)	NGFG_RS03020	0.9830							
2	NGO1379 (28-283 AA)	NGFG_RS07625	0.8485	0.7880	0.0079	-0.5581	25.8%	0.0247	100.0%	1.065
	cNGO0725 (1-109 AA)	NGFG_RS03865	0.7275							
3	NGO1158 (27-422 AA)	NGFG_RS06405	0.6706	0.6395	0.0159	-0.4293	27.5%	0.2348	95.5%	0.910
	NGO0182 (26-228 AA)	NGFG_RS00980	0.6083							
4	NGO0721 (22-337 AA)	NGFG_RS03820	0.5174	0.5348	0.0159	-0.4293	27.4%	0.2225	65.7%	-0.126
	NGO2105 (44-1468 AA)	NGFG_RS11765	0.5521							
5	cNGO1094 (1-398 AA)	NGFG_RS06105	0.4684	0.4505	0.0317	-0.1749	17.0%	-1.0628	24.4%	-1.560
	NGO1043 (22-114 AA)	NGFG_RS05785	0.4722							
	NGO2059 (22-522 AA)	NGFG_RS11450	0.4110							
6	cNGO1984 (59-216 AA)	NGFG_RS11045	0.3994	0.2746	0.2222	2.8920	18.5%	-0.8775	34.7%	-1.203
	cNGO1286 (1-943 AA)	NGFG_RS07120	0.3559							
	NGO1092 (1-649 AA)	NGFG_RS02670	0.0684							
7	cNGO0275 (28-1075 AA)	NGFG_RS01485	0.9642	0.9323	0.031	-0.1749	23.6%	-0.2472	65.1%	-0.148
	NGO0225 (25-628 AA)	NGFG_RS01205	0.9004							
8	cNGO1495 (25-912 AA)	NGFG_RS01410	0.7664	0.7745	0.0079	-0.5581	34.5%	1.0999	79.5%	0.354
	cNGO2093 (23-720 AA)	NGFG_RS11700	0.7825							
9	cNGO1392 (28-439 AA)	NGFG_RS07730	0.7772	0.6756	0.0556	0.2099	12.8%	-1.5819	68.2%	-0.039
	cNGO1585 (28-576 AA)	NGFG_RS07730	0.7772							
	cNGO2109 (23-809 AA)	NGFG_RS11785	0.4724							
10	cNGO1801 (22-792 AA)	NGFG_RS09970	0.4841	0.4144	0.0556	0.2099	20.8%	-0.5932	30.7%	-1.343
	cNGO0952 (26-922 AA)	NGFG_RS05315	0.3842							
	cNGO1715 (25-801 AA)	NGFG_RS09490	0.3750							
11	NGO1549 (35-289 AA)	NGFG_RS08575	0.8546	0.8340	0.0159	-0.4293	36.5%	1.3471	98.8%	1.025
	NGO0265 (44-346 AA)	NGFG_RS01435	0.8133							

Table S3B. EDEN prediction scores, and derivation of AUC and bactericidal Z-scores for *N. gonorrhoeae* H041 (WHO X)

Challenge Group	Recombinant Centroid Construct	Challenge strain: H041 (WHO X)								
		EDEN prediction score (of challenge homolog target proteins)			PROTECTION (*in vivo*)				PROTECTION (*in vitro*)	
	**Protein ID** "c" = centroid homolog is not a FA1090 strain protein	**Locus ID** (challenge homolog target protein)	**EDEN_pred_score_homolog**	**Group Mean (EDEN_pred_score_homolog)**	**AUC (log10 CFU)** [P-value, mann-whitney-test]	**Z-score normalized** (p-value)	**AUC (log10 CFU)** [% Median Reduction, Test group vs Ctrl group]	**Z-score normalized** (% median reduction)	**% bacterial killing**	**Z-score normalized**
1	cNGO1496 (1-693 AA)	KAE9495359	0.8014	0.8912	0.2222	-0.7450	16.1%	0.0457	38.6%	0.073
	cNGO0571 (21-598 AA)	KAE9498127	0.9810							
2	NGO1379 (28-283 AA)	KAE9499651	0.8531	0.4266	0.5476	0.5573	13.7%	-0.1051	21.1%	-0.590
	cNGO0725 (1-109 AA)	KAE9498848	0.0001							
3	NGO1158 (27-422 AA)	KAE9494197	0.6260	0.6172	0.4206	0.0490	3.2%	-0.7510	19.8%	-0.636
	NGO0182 (26-228 AA)	KAE9498999	0.6083							
4	NGO0721 (22-337 AA)	KAE9496551	0.4611	0.5067	0.5476	0.5574	-7.8%	-1.4322	21.5%	-0.574
	NGO2105 (44-1468 AA)	KAE9499234	0.5522							
5	cNGO1094 (1-398 AA)	KAE9495297	0.5157	0.4613	0.8413	1.7327	2.0%	-0.8264	21.7%	-0.566
	NGO1043 (22-114 AA)	KAE9493985	0.4722							
	NGO2059 (22-522 AA)	KAE9499767	0.3961							
6	cNGO1984 (59-216 AA)	KAE9499842	0.3818	0.4019	0.2222	-0.7450	29.0%	0.8424	74.1%	1.413
	cNGO1286 (1-943 AA)	KAE9499561	0.3682							
	NGO1092 (1-649 AA)	KAE9495295	0.4555							
7	cNGO0275 (28-1075 AA)	KAE9498849	0.9771	0.4995	0.6905	1.1292	7.2%	-0.5035	13.5%	-0.873
	NGO0225 (25-628 AA)	KAE9498897	0.0219							
8	cNGO1495 (25-912 AA)	KAE9495360	0.8043	0.7880	0.2222	-0.7450	13.2%	-0.1358	12.6%	-0.907
	cNGO2093 (23-720 AA)	KAE9499247	0.7716							
9	cNGO1392 (28-439 AA)	KAE9496065	0.7715	0.6832	0.2222	-0.7450	15.8%	0.0292	26.5%	-0.384
	cNGO1585 (28-576 AA)	KAE9496065	0.7715							
	cNGO2109 (23-809 AA)	KAE9499232	0.5065							
10	cNGO1801 (22-792 AA)	KAE9499320	0.4841	0.2971	0.5476	0.5573	23.6%	0.5101	68.0%	1.180
	cNGO0952 (26-922 AA)	KAE9496328	0.0322							
	cNGO1715 (25-801 AA)	KAE9499718	0.3750							
11	NGO1549 (35-289 AA)	KAE9496098	0.8664	0.8399	0.0079	-1.6027	53.0%	2.3321	86.1%	1.863
	NGO0265 (44-346 AA)	KAE9498859	0.8133							
