# Supplementary material for: Preclinical efficacy of a cell division protein candidate gonococcal vaccine identified by artificial intelligence
Source: mBio. 2023 Oct 31;14(6):e02500-23. doi: 10.1128/mbio.02500-23 (PMC10746169; doi:10.1128/mbio.02500-23)
Supplement: Table S4 — Characteristics of the 50 strains of N. gonorrhoeae used in Fig. 9 and Fig. S5. [file mbio.02500-23-s0009.pdf]

Supplemental Table S1. Characteristics of the 50 strains of *N. gonorrhoeae* used in Figure 9 and Supplemental Figure S5

Strain	Relevant characteristics	Ref
WHO F	NCTC no. 13477; PorB1a; MLST – ST10934; NG-MAST – ST3303	(1)
WHO G	NCTC no. 13478; PorB1a; MLST – ST1903; NG-MAST – ST621	(1)
WHO K	NCTC no. 13479; PorB1b; MLST – ST7363; NG-MAST -ST1424	(1)
WHO L	NCTC no. 13480; PorB1b; MLST - ST1590; NG-MAST – ST1422	(1)
WHO M	NCTC no. 13481; PorB1b; MLST – ST7367; NG-MAST – ST3304	(1)
WHO N	NCTC no. 13482; PorB1a; MLST – ST1583; NG-MAST – ST556	(1)
WHO O	NCTC no. 13483; PorB1b; MLST – ST1902; NG-MAST – ST495	(1)
WHO P	NCTC no. 13484; PorB1b; MLST – ST8127; NG-MAST – ST3305	(1)
WHO U	NCTC no. 13817; PorB1b; MLST – ST7367; NG-MAST – ST2382	(1)
WHO V	NCTC no. 13818; PorB1b; MLST – ST10314; NG-MAST – ST8927	(1)
WHO W	NCTC no. 13819; PorB1b; MLST – ST7363; NG-MAST – ST835	(1)
WHO X	NCTC no. 13820; PorB1b; MLST – ST7363; NG-MAST – ST4220	(1)
WHO Y	NCTC no. 13821; PorB1b; MLST – ST1901; NG-MAST – ST1407	(1)
WHO Z	NCTC no. 13822; PorB1b; MLST – ST7363; NG-MAST – ST4015	(1)
FA1090	PorB1b; MLST - ST1899; NG-MAST – ST773	(2)
FA19	PorB1a MLST – ST1892; NG-MAST – ST13745	(3)
MS11	PorB1b MLST – ST6959; NG-MAST - ST4813	(3)
F62	PorB1b MLST – ST1900; NG-MAST – ST915	(2)
252	PorB1a	(4)
NJ1 (UMNJ1)	PorB1b; MLST – ST11969; NG-MAST – ST3366	(5)
NJ11 (UMNJ11)	PorB1b; MLST – ST 11173; NG-MAST – ST270	(5)
NJ13 (UMNJ13)	PorB1a MLST – ST7367; NG-MAST – ST19720	(6)
NJ15 (UMNJ15)	PorB1b; MLST - ST1583; NG-MAST – ST19440	(5)
NJ19 (UMNJ19)	PorB1b; MLST - ST11984; NG-MAST - ST19439	(5)
NJ24 (UMNJ24)	PorB1a MLST - ST10633; NG-MAST - ST19721	(6)
NJ26 (UMNJ26)	PorB1b; MLST – ST1580; NG-MAST - ST11998	(5)
NJ27 (UMNJ27)	PorB1b; MLST – ST7827; NG-MAST – ST2318	(5)
NJ31 (UMNJ31)	PorB1a MLST – ST7356; NG-MAST - ST568	(6)
NJ36 (UMNJ36)	PorB1b; MLST – ST1600; NG-MAST – ST1766	(5)
NJ44 (UMNJ44)	PorB1b; MLST – ST1901; NG-MAST - ST8736	(5)
NJ48 (UMNJ48)	PorB1b; MLST – ST1583; NG-MAST – ST1053	(5)
NJ60 (UMNJ60)	PorB1b; MLST – ST1600; NG-MAST – ST3289	(5)
NJ62 (UMNJ62)	PorB1a MLST – ST1588; NG-MAST - ST12508	(6)
NJ63 (UMNJ63)	PorB1a MLST – ST1588; NG-MAST - ST12508	(6)
NJ68 (UMNJ68)	PorB1a MLST – ST7365; NG-MAST - ST18351	(6)
NJ69 (UMNJ69)	PorB1a MLST – ST7365; NG-MAST - ST18351	(6)
NJ99 (UMNJ99)	PorB1a: MLST – ST7822; NG-MAST - ST10335	(6)
OC7	PorB1b; NG-MAST – ST8535	(7)
OC14	PorB1b; NG-MAST – ST3307	(7)
SD3	PorB1b; NG-MAST – ST2400	(7)
SD5	PorB1b; NG-MAST – ST1407	(7)

SD8	PorB1b; NG-MAST – ST2400	(7)
SD15	PorB1b; NG-MAST – ST1407	(7)
SF2	PorB1b; NG-MAST – ST1407	(7)
SF6	PorB1b; NG-MAST – ST8424	(7)
SF7	PorB1b; NG-MAST – ST8481	(7)
WR220	PorB1b	(8)
1291	PorB1b; MLST – ST 8422; NG-MAST - 19075	(9)
334	PorB1b	(10)
03701 Cx	PorB1b	(6)
PID LS	PorB1b	(10)
PID1	PorB1b; MLST 10154; NG-MAST - 19316	(10)
PID8	PorB1b	(10)
PID02601	PorB1b	(6)
PID333	PorB1b	(10)
PID6860	PorB1a	(10)
PID02201	PorB1b	(6)
PID11	PorB1b	(10)
24-1	PorB1b; MLST – ST8418, NG-MAST - 19315	(10)
UU1	PorB1a	(11)

References

1. Unemo M, Golparian D, Sanchez-Buso L, Grad Y, Jacobsson S, Ohnishi M, Lahra MM, Limnios A, Sikora AE, Wi T, Harris SR. 2016. The novel 2016 WHO Neisseria gonorrhoeae reference strains for global quality assurance of laboratory investigations: phenotypic, genetic and reference genome characterization. J Antimicrob Chemother 71:3096-3108.
2. Dillard JP, Seifert HS. 2001. A variable genetic island specific for Neisseria gonorrhoeae is involved in providing DNA for natural transformation and is found more often in disseminated infection isolates. Mol Microbiol 41:263-77.
3. Carbonetti NH, Simnad VI, Seifert HS, So M, Sparling PF. 1988. Genetics of protein I of Neisseria gonorrhoeae: construction of hybrid porins [published erratum appears in Proc Natl Acad Sci U S A 1989 Feb;86(4):1317]. Proc Natl Acad Sci U S A 85:6841-5.
4. McQuillen DP, Gulati S, Ram S, Turner AK, Jani DB, Heeren TC, Rice PA. 1999. Complement processing and immunoglobulin binding to Neisseria gonorrhoeae determined in vitro simulates in vivo effects. J Infect Dis 179:124-35.
5. Shaughnessy J, Gulati S, Agarwal S, Unemo M, Ohnishi M, Su XH, Monks BG, Visintin A, Madico G, Lewis LA, Golenbock DT, Reed GW, Rice PA, Ram S. 2016. A Novel Factor H-Fc Chimeric Immunotherapeutic Molecule against Neisseria gonorrhoeae. Journal of immunology 196:1732-40.
6. Shaughnessy J, Chabeda A, Tran Y, Zheng B, Nowak N, Steffens C, DeOliveira RB, Gulati S, Lewis LA, MacLean J, Moss JA, Wycoff KL, Ram S. 2022. An optimized Factor H-Fc fusion protein against multidrug-resistant *Neisseria gonorrhoeae*. Front Immunol.
7. Gose S, Nguyen D, Lowenberg D, Samuel M, Bauer H, Pandori M. 2013. Neisseria gonorrhoeae and extended-spectrum cephalosporins in California: surveillance and molecular detection of mosaic penA. BMC Infect Dis 13:570.

8. Apicella MA, Westerink MAJ, Morse SA, Schneider H, Rice PA, Griffiss JM. 1986. Bactericidal antibody response of normal human serum to the lipooligosaccharide of *Neisseria gonorrhoeae*. J Infect Dis 153:520-526.
9. Dudas KC, Apicella MA. 1988. Selection and immunochemical analysis of lipooligosaccharide mutants of *Neisseria gonorrhoeae*. Infect Immun 56:499-504.
10. Kasper DL, Rice PA, McCormack WM. 1977. Bactericidal antibody in genital infection due to *Neisseria gonorrhoeae*. J Infect Dis 135:243-251.
11. Wetzler LM, Blake MS, Gotschlich EC. 1988. Characterization and specificity of antibodies to protein I of Neisseria gonorrhoeae produced by injection with various protein I- adjuvant preparations. J Exp Med 168:1883-97.
