# Supplementary material for: Preclinical efficacy of a cell division protein candidate gonococcal vaccine identified by artificial intelligence
Source: mBio. 2023 Oct 31;14(6):e02500-23. doi: 10.1128/mbio.02500-23 (PMC10746169; doi:10.1128/mbio.02500-23)
Supplement: Table S5 — Strains used to identify centroid sequences of antigens. [file mbio.02500-23-s0010.pdf]

Table S5. Strains used to identify centroid sequences of antigens

STRAINS (CENTROID IDENTIFICATION)					
#	Strain name	#	Strain name	#	Strain name
1	FA1090	51	GCGS144	101	PID1
2	1291	52	GCGS146	102	PID18
3	35_02	53	GCGS147	103	PID332
4	ALB_2011_01_02	54	GCGS150	104	SK33414
5	ALB_2011_03_03	55	GCGS158	105	SK_92_679
6	ALB_2011_04_03	56	GCGS162	106	SK_93_1035
7	ATCC49226	57	GCGS174		
8	ATL_2011_01_21	58	GCGS176		
9	ATL_2011_01_25	59	GCGS178		
10	ATL_2011_05_13	60	GCGS183		
11	DGI2	61	GCGS192		
12	F62	62	GCGS193		
13	FA19	63	GCGS195		
14	GCGS001	64	GCGS197		
15	GCGS006	65	GCGS198		
16	GCGS011	66	GCGS201		
17	GCGS018	67	GCGS202		
18	GCGS021	68	GCGS203		
19	GCGS030	69	GCGS204		
20	GCGS036	70	GCGS207		
21	GCGS039	71	GCGS209		
22	GCGS044	72	GCGS210		
23	GCGS045	73	GCGS211		
24	GCGS047	74	GCGS212		
25	GCGS048	75	GCGS213		
26	GCGS049	76	GCGS215		
27	GCGS050	77	GCGS216		
28	GCGS061	78	GCGS217		
29	GCGS062	79	GCGS224		
30	GCGS069	80	GCGS226		
31	GCGS077	81	GCGS231		
32	GCGS079	82	GCGS234		
33	GCGS082	83	MIA_2011_03_09		
34	GCGS084	84	MIA_2011_03_10		
35	GCGS086	85	MIA_2011_05_10		
36	GCGS089	86	MIA_2011_05_15		
37	GCGS100	87	MIA_2011_05_16		
38	GCGS102	88	MU_NG12		
39	GCGS103	89	MU_NG15		
40	GCGS104	90	MU_NG17		
41	GCGS114	91	MU_NG19		
42	GCGS115	92	MU_NG20		
43	GCGS116	93	MU_NG25		
44	GCGS127	94	MU_NG3		
45	GCGS128	95	MU_NG4		
46	GCGS134	96	MU_NG5		
47	GCGS135	97	MU_NG6		
48	GCGS137	98	MU_NG8		
49	GCGS139	99	NOR_2011_03_06		
50	GCGS141	100	NYC_2011_05_07		
